# Water‐Splitting “Without Water”: Splitting of the Crystallised Water of Hydrated Salts

**DOI:** 10.1002/anie.202520018

**Published:** 2025-12-06

**Authors:** Klara Rüwe, Shirui Wang, Tom Bookholt, Julia Brune, Hannelore Schmidt, Marius Behnecke, Svea Petersen, Claudia Heß, Alex M. Ganose, Helmut Schäfer

**Affiliations:** ^1^ Department of Biology and Chemistry The Electrochemical Energy and Catalysis Group University of Osnabrück Barbarastrasse 7 49076 Osnabrück Germany; ^2^ Department of Chemistry Molecular Sciences Research Hub White City Campus Imperial College London Wood Lane London W12 0BZ UK; ^3^ Faculty of Engineering and Computer Science Laboratory for Organic Chemistry and Polymer Chemistry University of Applied Sciences Osnabrück P.O. Box 1940 49009 Osnabrück Germany; ^4^ Department of Biology and Chemistry Physical Chemistry University of Osnabrück Barbarastrasse 7 49076 Osnabrück Germany

**Keywords:** Computational chemistry, Electrocatalysis, Electrochemistry, Energy conversion

## Abstract

In contrast to the liquid phase of water, the structural molecular realities of H_2_O molecules embedded in crystallised salts vary little and can be precisely determined. However, this water, which is neither in the liquid nor in the gas phase, has not yet been used in water electrolysis. Here, we demonstrate that water electrolysis can be achieved without the addition of (liquid) water through experiments with a wide range of hydrated salts and organic solvent suspensions. We obtain an excellent correlation between the position of the FTIR O─H stretch vibration of the hydrated salts and the onset of the OER potential from CV measurements. Together with first‐principles density functional theory calculations, we demonstrate that intramolecular bonds in crystallised water can be effectively controlled through choice of the inorganic salt. Targeted manipulation of the bonds in the H_2_O molecule is a promising new approach to more efficient hydrogen production.

## Graphical abstract

Unlike liquid water, the structural and molecular properties of water molecules embedded in hydrated salts are subject to strong influences and vary little. This makes them potential candidates for the water splitting reaction. Here, we present the first example of the electrochemical decomposition of crystallised water.

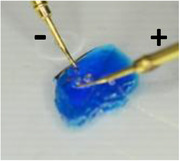

## Introduction

The ever‐growing number of people on our planet increases greenhouse gas emissions from livestock and industrial processes, causing environmental and energy problems^[^ ^1^, ^2^ ^]^ Splitting water into O_2_ and H_2_ using sunlight is a clean, sustainable solution to many of society's energy (storage and conversion) problems as solar energy and water are generally abundant, and hydrogen is a clean fuel3. Water electrolysis, when powered by green electricity, is a CO_2_ footprint‐free realisation of water splitting, but its economic and ecological value depends, among other things, on its efficiency, which in turn is directly determined by the sum of the overpotentials that occur at both electrodes.^[^ ^3^ ^]^ The question of how to reduce over voltages has been at the centre of scientific debate and has filled review articles and research papers alike.^[^ ^4^, ^5^, ^6^, ^7^, ^8^, ^9^, ^10^, ^11^, ^12^] The vast majority of the research effort is devoted, directly or indirectly, to improving electrodes to make both half‐cell reactions more efficient.^[^ ^4^, ^5^, ^13^ ^]^

Water, the main component of electrolytes currently used for water electrolysis, plays a critical role in many biological and chemical processes due to its unique properties.^[^ ^14^ ^]^ Some of the unique fundamental physical properties of the condensed phase have been attributed to the cooperative hydrogen bonding network of multiple water molecules.^[^ ^15^, ^16^ ^]^ When four H‐ bonded H_2_O molecules organise in tetrahedral coordination it is defined as an ice‐like water structure. Based on the bond conservation principle, there is a fundamental relationship between the intermolecular and intramolecular structure of water. As the hydrogen bond strength increases (i.e., the intermolecular O─H bonds), the strength of the intramolecular O─H bonds decreases, leading to an inverse correlation between the frequency of the O─H stretch vibration and the hydrogen bond strength.^[^ ^17^ ^]^ Whenever an organic or inorganic compound is added to water (naturally leading to a reduced water content), it will influence the hydrogen bonding network and support or hinder the water electrolysis reaction. Water splitting in “confined” water systems, i.e., in electrolytes with reduced water content has already been studied for example by using ionic liquids^[^ ^18^, ^19^ ^]^ or “water‐in‐salt‐electrolytes”.^[^ ^20^, ^21^, ^22^ ^]^ However, as far as we know, none of the published approaches have improved or aimed to improve the efficiency of water electrolysis by activating the intramolecular bonding of water molecules. Moreover, none of the above studies investigated a possible relationship between water molecule structural molecular realities and aqueous electrolyte readiness to support water electrolysis.

Thus, Suo et al. reported on a highly concentrated aqueous electrolyte consisting of lithium bis (trifluoromethane sulfonyl) imide (molality > 20 m).^[^ ^20^ ^]^ In this system, the hydrogen bonding network is significantly weakened, leading to more stable intramolecular O─H bonds – and therefore to a significantly reduced electrochemical activity of water, which allows the electrochemical stability window of water to be extended to 3 V. This has become a popular strategy for replacing the flammable and expensive organic electrolytes in batteries with cheaper water‐based electrolytes.^[^ ^21^, ^22^ ^]^ Hydrogen evolution catalysis has been investigated in diethylammonium formate ionic liquid electrolyte by Li et al. and a reduction of the HER overpotential in comparison to K_2_SO_4_ based electrolyte was found 18. It has been suggested that the slight improvement in HER efficiency is due to the interaction of the amine groups with the Pt‐based catalyst.

We have recently shown that the propensity of water molecules to split into their cleavage products, oxygen and hydrogen, does indeed vary with their structural‐molecular reality, which in turn was found to vary in binary solutions of 1,4‐dioxane in water,^[^ ^17^ ^]^ as well as in suspensions of NiO in sulphuric acid.^[^ ^23^ ^]^ However, sometimes experimental efforts such as those described above to activate (weaken) the bonds in water molecules for an intended cleavage are not even necessary. For example, when water molecules are “embedded” in inorganic salts (hydrated salts) to form crystallised water (water of crystallisation), the molecules are under exceptionally strong influence, as shown by neutron diffraction studies.^[^ ^24^, ^25^, ^26^ ^]^ Again, based on the bond valence model, when cations, for example, interact with oxygen atoms of water molecules, the intramolecular O─H bonds are lengthened because there is less bonding strength available.^[^ ^24^ ^]^ This also explains the lengthening of the intramolecular O─H bonds if the cation charge increases. Depending on the nature of the salt, there may be either a lengthening or shortening of the (intramolecular) O─H bonds in crystallised water relative to the corresponding bond lengths in liquid water. The exclusive use of activated water molecules in water electrolysis experiments, which are present as water of crystallisation, ideally presupposes that no “loose” water is added, i.e., that the crystallised water can be split directly, e.g., in hydrated salt/organic solvent suspensions, which to our knowledge has never been proven. In this work, we demonstrate the direct cleavage of water of crystallisation for the first time, through the study of suspensions of toluene and hydrated salts. We further highlight that in some hydrated salt/organic solvent systems embedded water molecules are even more active towards water electrolysis than those of liquid water. Through a combination of first‐principles calculations, FTIR spectroscopic studies, and cyclic voltammograms, we reveal the critical role of bond length manipulation as responsible for determining the onset of oxygen evolution potential.

It is clear that the choice of an organic solvent‐based electrolyte with a high solids content (the basis of our approach), rather than a clear, classic water‐based electrolyte, has disadvantages, such as lack of transferability to existing electrolyser designs and reduced conductivity. Therefore, in addition to direct cleavage of the water of crystallisation, we have investigated the extent to which dissolved salt can be used to manipulate the intramolecular bonding of water molecules in aqueous solutions. Salts discussed in this paper are considered strong electrolytes, i.e., they undergo complete dissociation. The electric field of the ions influences the surrounding water molecules (first sphere of coordination).^[^ ^27^, ^28^ ^]^ However, the effect on the water molecules goes beyond this first hydration shell, and the hydrogen bonding network of water as a whole (bulk water) is affected when salts are added, resulting in either a supported or disturbed ice‐like water structure.^[^ ^28^, ^29^ ^]^ Salts can therefore be divided into water structure enhancers (makers), which strengthen the ice‐like water structure and weaken the intramolecular O─H bonds, and water structure disruptors (breakers), which weaken the ice‐ like water structure and therefore strengthen the intramolecular O─H bonds.^[^ ^28^, ^29^, ^30^ ^]^ Surprisingly, the influence of water structure‐making salts on the ability of aqueous solutions to support water electrolysis has not yet been investigated. Indeed, as proposed, dissolving a water structure‐making salt in sulphuric acid led to a significant reduction in the cell voltage for water electrolysis, confirming the practicality of our approach also in aqueous solutions. Our work therefore aims to have a broad impact on the design of scalable routes for efficient hydrogen production.

## Results and Discussion

### Splitting of the Crystallised Water of Hydrated Salts

**Figure 1:**
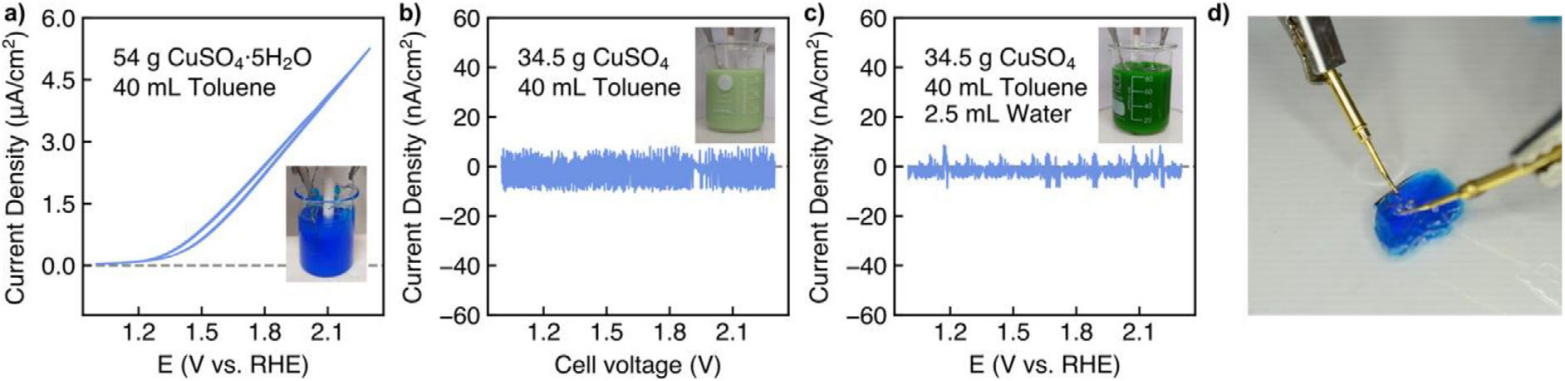
Splitting of crystallised water in CuSO_4_·5 H_2_O. Cyclic voltammetry measurements, current density versus cell voltage plots performed on a slurry consisting of a) 54 g CuSO_4_·5 H_2_O and 40 mL toluene, b) 34.5 g CuSO_4_ and 40 mL toluene, and c) 34.5 g of CuSO_4_, 40 mL of toluene and 2.5 mL of a pH 7 buffer solution, respectively. Inset pictures show the corresponding slurries. d) Photograph showing the decomposition of a CuSO_4_·5 H_2_O crystal, 9 x 6 x 5 mm in size, under an electric current in toluene.

In a first set of experiments, we prepared toluene/CuSO_4_·5 H_2_O slurries and determined the onset of oxygen evolution potential by performing cyclic voltammetry measurements using a three‐electrode setup consisting of a Pt working electrode (WE) as anode, a Pt counter electrode (CE) and a reversible hydrogen electrode as the reference electrode. Platinum is the electrode material of choice when it comes to unmasking electrolyte effects^[^ ^17^ ^]^; subtle effects of the electrolyte can only be detected if it is ensured that the electrode does not undergo any changes during the CV experiments. The best way to ensure this is to use Pt for both electrodes (WE and CE). The use of 54 g (216.3 mmol) of CuSO_4_·5 H_2_O in 40 mL of toluene resulted in an intensely blue, extremely viscous slurry (inset in Figure 1a) into which the electrodes were simply placed (without stirring). The CV, (a video showing the recording is given in the Supporting Information Video 1) of this slurry shows a typical curve resulting from the onset of oxygen evolution (Figure 1a). This is the first time that the splitting of water of crystallisation has been demonstrated to our knowledge. The curve course also allows a very accurate determination of the onset potential due to the extremely sharp rise in current density.

Onset potential is often seen as a simple and easy measure parameter that indicates the relative activity of a material. However, although widely reported, there is no clear or universally accepted definition of “onset potential”.^[^ ^31^ ^]^ The most common way of defining the onset potential is as the point at which the current density exceeds a certain value, which is problematic if the electrolyte composition varies significantly with dramatic changes in conductivity within a series of tests. In addition, this method is specifically *“sensitive to the surface catalyst loading and the specific surface area of the catalytic material”* 31. We have therefore chosen the tangent method, a detailed description of which is given in the Supporting Information. OER begins in a hydrated CuSO_4_/toluene mixture at 1.41 V versus RHE (Figure S1). To confirm that the current density seen in the CV (Figure 1a) is based on OER, we carried out the electrolysis of a water‐free CuSO_4_/toluene‐based slurry (Figure 1b) as well as the electrolysis of this slurry after the addition of liquid water (Figure 1c). Due to the very low conductivity of the toluene‐based mixture when using anhydrous salt, a CV could not be measured because the potentiostat reaches its voltage limit (the potential of the WE cannot be set to the values shown on the abscissa in the CV (Figure 1a). Instead of the potential of the WE relative to the RE, the cell voltage is shown when CuSO_4_/toluene was subjected to the CV measurement (Figure 1b). The current density shown on the ordinate axis of both plots (Figure 1b, 1c) is very close to zero, confirming that no water splitting has taken place. However, this finding strongly suggests that water electrolysis was successful when a slurry of toluene/CuSO_4_·5 H_2_O was subjected to the electrolysis experiments (Figure 1a).

Using our standard setup, it is not possible to electrochemically decompose toluene at moderate potentials, as the current density/cell voltage diagram clearly rules out this hypothesis (Figure S2). This was confirmed by further testing (see below), and is consistent with other reports.^[^ ^32^ ^]^ Unfortunately, we could not find a supporting electrolyte that dissolved well in toluene. A low‐concentration solution of tetra‐n‐butylammonium hexafluorophosphate in toluene (200 mg in 60 ml of toluene) was prepared, the CV's of which shown in Figure S3 as well as the chronoamperometry results confirm the electrochemical stability of toluene (Figures S4–S7).

We have not been able to obtain reasonable electronic impedance spectroscopy (EIS) results with pure toluene. However, an EIS study carried out on a CuSO4·5 H_2_O /toluene slurry gave a reasonable result, a Nyquist plot of which is shown in Figure S8, allows an estimation of the electrolyte and polarisation resistances (Re = 3400 Ω; Rp = 150 kΩ).

To rule out the possibility that traces of water could escape from CuSO_4_·5 H_2_O after contact with toluene and cause the anodic current wave seen in Figure 1a, a further experiment was carried out. The water content of toluene was determined by Karl Fischer titration (see Supporting Information for details) after 40 mL of toluene had been in contact with 54 g of CuSO_4_·5 H_2_O (the exact composition of the electrolyte used to record the CV of Figure 1a) for 2 h. The water content was found to be 72.8 ppm, which is at the level of the water content in unused toluene (70.3 ppm). The current density plotted against the cell voltage recorded with the solution obtained after filtration of this experiment shows no evidence of oxygen evolution (the arithmetic mean of the current density amounts to 8.4·10−3 µA/cm2; Figure S9), clearly suggesting that only the solid compound (CuSO_4_·5 H_2_O) in the salt/toluene slurry (inset of Figure 1a) is responsible for the current seen in the CV (Figure 1a). In order to further manifest this only reasonable interpretation of the observations, larger, mm sized CuSO_4_·5 H_2_O crystals were grown and directly contacted with gold electrodes (Supporting Information). When an electric current is passed through one of these crystals, it is clearly visible that gas is formed on both electrodes under the toluene (Figure 1d, Videos 2, 3, Supporting Information). In addition, we repeated the experiment with the bare crystal in air (i.e., without toluene). Once again, an electric current was produced and the crystal decomposed. This clearly suggests that it is not the toluene that decomposes in electrolysis experiments performed with salt/toluene slurries (see Figure 1a). These findings shed a great deal of light on the exact mechanism of electrolysis and strongly suggests that the electrolysis of crystallised water of hydrated salts in salt/organic solvent suspensions, as seen for example in CV experiments (Figure 1a), occurs by decomposition of crystals arranged in rows. The diffusion of water out of the surface of a hydrated CuSO_4_ crystal under electrolysis conditions was not confirmed, as applying an electric field of *E* = 1.4 V mm^−1^ to an mm‐sized crystal while carrying out FTIR spectroscopy on its surface did not result in any changes (see Figure S10).

We have studied the gas released from a CuSO4·5 H2O crystal during electrolysis under toluene using a gas chromatography‐mass spectrometry (GC‐MS) headspace approach (Supporting Information).Intensive formation of oxygen gas in the headspace was confirmed after the crystal was subjected to electrolysis for 200s (Figures S11–S15).

Unlike anhydrous CuSO_4_, which showed no signs of decomposition under an electric current (Figure 1b), CuSO_4_·5 H_2_O did (Figure 1d). This was also confirmed by conductivity tests; we pressed tablets of anhydrous CuSO_4_ and CuSO_4_·5 H_2_O, fitted them with gold contacts and subjected them to a conductivity test. While the anhydrous form of the salt was found to be non‐conductive, CuSO_4_·5 H_2_O was conductive (Figure S16).

Similar to hydrated CuSO_4_, we were able to split crystallised water in Al_2_(SO_4_)_3_·18 H_2_O using a toluene slurry (Figure S17). However, due to the generally very low conductivity of toluene slurries, we encountered some problems in obtaining reasonable CV results with other salts when using toluene, so we switched to DMSO‐based electrolytes.

DMSO itself (as toluene) was found to be electrochemically inert (Figure S18) under the chosen conditions, and whereas pure DMSO exhibited an enormous electrolyte resistance (*Re*) (*Re* = 3300 Ω) Figure S19), the addition of hydrated salts to DMSO drastically reduced it (CuSO_4_·5 H_2_O: *Re* = 130 Ω, Co(NO*_3_*)_2_·6 H_2_O: *Re* = 33 Ω, FeSO_4_·7 H_2_O: *Re* = 380 Ω, NiSO_4_·6 H_2_O: *Re* = 280 Ω, Fe(NO_3_)_3_·9 H_2_O: *Re* = 50 Ω, Fe_2_(SO_4_)_3_·x H_2_O: *Re* = 1035 Ω) as shown by EIS experiments (Figure S20a–g). In addition, the Nyquist plots show a significant reduction of the polarisation resistance *Rp*, e.g., from 124 kΩ (DMSO) to 60 Ω, determined for a suspension of 20 g CuSO_4_·5 H_2_O in 22 mL DMSO, clearly confirming the completely new nature of the electrolyte that occurs when the hydrated salt is added to the organic solvent.

### A Comparison of Water Splitting Achieved with Different Hydrated Salt/Dimethyl Sulfoxide Suspensions

We next carried out water splitting experiments with suspensions consisting of different hydrated salt/DMSO mixtures. We posit that a comparison of cyclic voltammetry (CV) measurements for different salt/DMSO compositions should reveal the influence of the molecular environment of the H_2_O molecule on the onset potential. This is only correct and reliable if the influence of the working electrode on the onset potential of oxygen evolution is the same for each CV measurement performed, i.e., if the electrode does not change during the CV experiments. The cyclic voltammetry studies therefore had to be carried out carefully following a strict protocol (see Experimental section).

There are a few limitations to consider. As we later plan to evaluate the structural environment of the H_2_O molecules through FTIR analysis, the selection of salts was performed with this in mind. Whilst sulphates and nitrates typically do not show absorption bands that may overlap with the O─H stretch vibration of water molecules, acetates are not suitable due to the C─H valence mode. From an electrochemical point of view, chlorides can cause problems due to possible chlorine evolution reactions. We ensure that a suspension is produced in each case (Figures S21–S26, i.e., the compositions of the mixtures (salt to DMSO ratio) vary slightly, as does the solubility of the (hydrated) salt in DMSO (see Supporting Information). However, it transpired that the precise water (or salt) content solely influences the formation of a suspension, rather than the onset of oxygen evolution potential.

**Figure 2:**
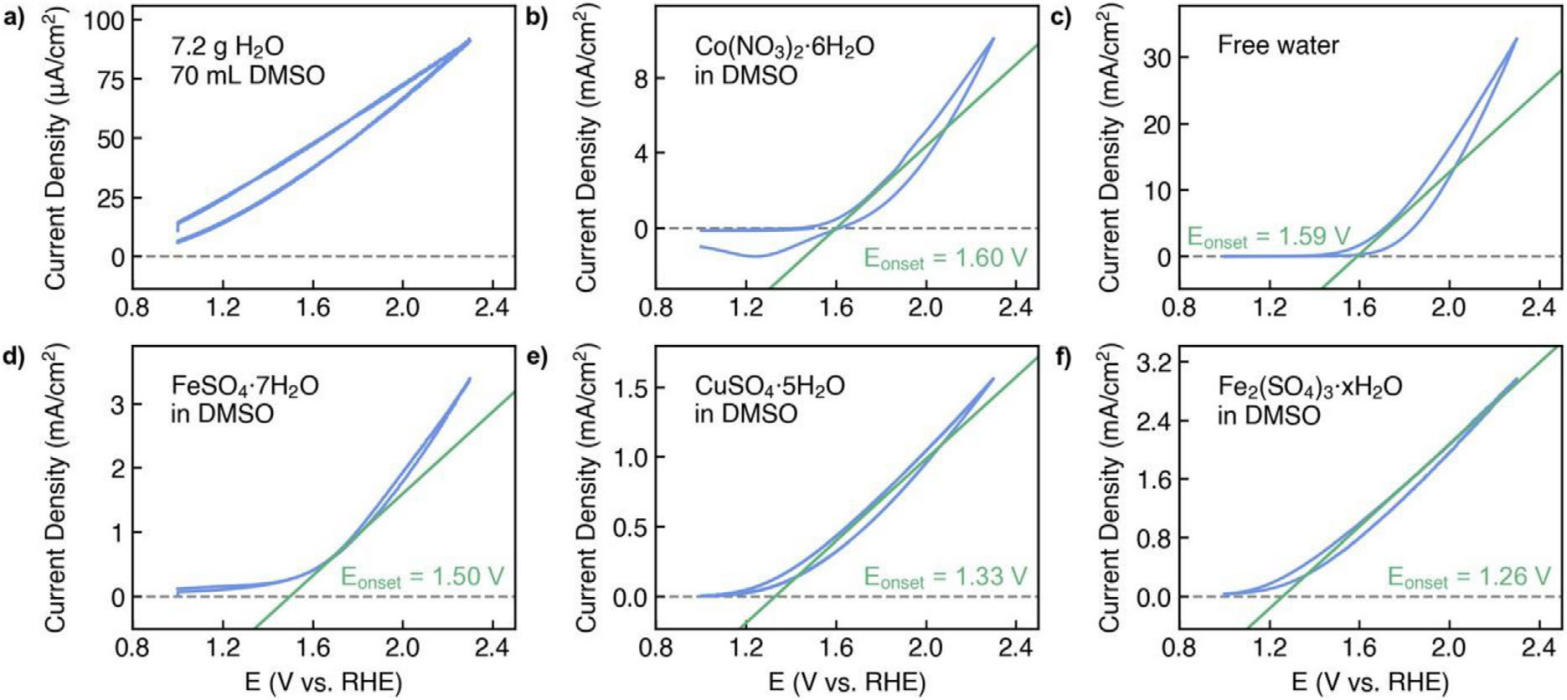
Cyclic voltammetry measurements performed with a) DMSO/pH 7 corrected 0.1 molar (K_2_HPO_4_/KH_2_PO_4_) buffer solution, b, d–f) hydrated salt/DMSO suspensions and c) pH 7 corrected 0.1 molar (K_2_HPO_4_/KH_2_PO_4_) buffer solution. Platinum was chosen as WE and CE; electrode area: 1 cm2 (WE), 3 ×4 cm geometric area (CE). Scan rate: 10 mV/s.

In Figure 2, we demonstrate water splitting across a broad range of hydrated salts. CVs derived from binary water/DMSO solutions show no clear evidence of the onset of water splitting (Figure 2a). This is consistent with earlier findings showing that adding an organic solvent to an aqueous binary system reduces the readiness of water molecules to decompose, as the intramolecular bonds are strengthened.^[^ ^17^ ^]^

Meanwhile, all other tested solutions consisting of DMSO‐based suspensions of CuSO_4_·5 H_2_O, FeSO_4_·7 H_2_O, NiSO_4_·6 H_2_O, Fe_2_(SO_4_)_3_·x H_2_O, Co(NO_3_)·6 H_2_O and Fe(NO_3_)_3_·9 H_2_O together with pure pH 7 electrolyte clearly showed the onset of the oxygen evolution reaction at different onset potentials (depending on the salt used) (Figures 2b–2f, S25, and S26). As can be seen from the tangents added to each CV plot, the onset of the oxygen evolution potential shifts between 1.21 V versus RHE (Fe_2_(SO_4_)_3_·x H_2_O) and 1.60 V versus RHE (Co(NO_3_)_2_·6 H_2_O). These results suggest that due to the different structural–molecular realities of the crystallised water molecules, these have different propensities to support the cleavage reaction, resulting in different OER onset potentials.

The surface of the Pt electrode did not change when using as oxygen evolving electrode in the CV measurements as can be seen from energy‐dispersive X‐ray spectroscopy (EDS) experiments carried out with the unused Pt, the Pt anodes after being used for CV tests according to Figure 2b, e, respectively (Figures S27–S29). Depending on the salt used, cathodic deposition of metal, i.e., plating on the cathode may occur (See for instance Figure S24). In addition, in particular, video 3 shows that cathodic deposition of Cu takes place simultaneously with hydrogen evolution.

The structural molecular states of water molecules in hydrated salts is influenced by the nature and oxidation state of the metal cation, the nature of the anion in the salt and the level of hydration. Most of the salt/DMSO mixtures tested had an onset potential below that obtained for pure pH 7 corrected 0.1 molar (KH_2_PO_4_/K_2_HPO_4_) buffer solution (referred to as free water, see Figure 2c), showing that water molecules embedded as crystallised water in hydrated salts are more active in water splitting than those in (liquid) water itself. The water molecules that represent the crystallised water in Fe_2_(SO_4_)_3_·H_2_O are subject to a unique and strong influence, as several effects converge (Fe^3^⁺ cations, SO_4_^2−^ anions and a low level of hydration). Consequently, these molecules exhibit exceptional activity in splitting (Figure 2f).

**Figure 3:**
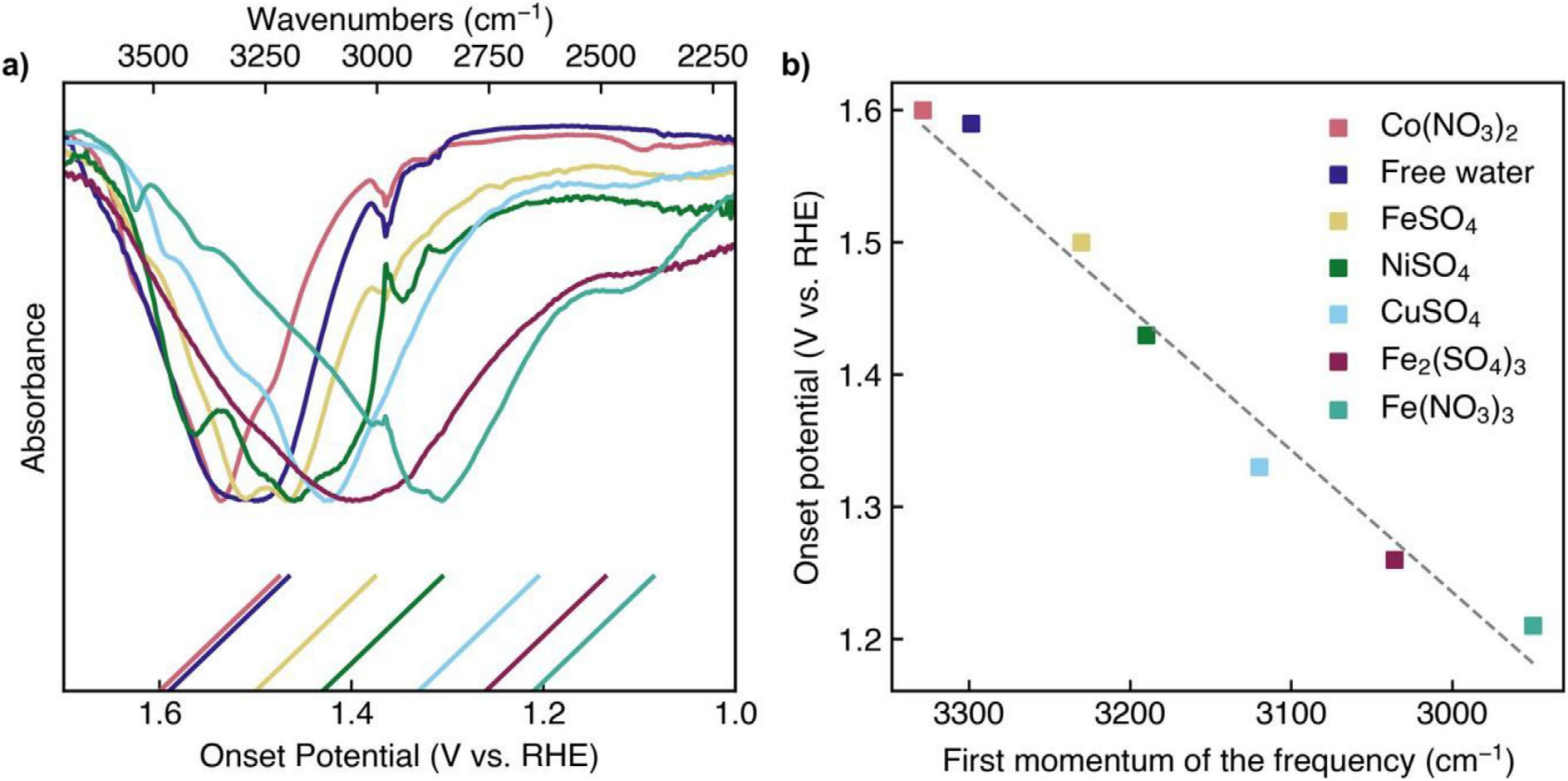
Correlation between FTIR and electrochemical results. a) Plot of wave numbers versus onset potential. The lower abscissa shows the onset potentials of different hydrated salt/DMSO suspensions as derived by the tangent method (45° tangents shown on the button), the upper abscissa shows the wave number of the FTIR spectra of the hydrated salts. Water has been omitted from the salt formulae for clarity. The absorbance of the salts has been normalised and is not to scale. b) Correlation between the onset of the OER potential derived from DMSO/hydrated salt suspensions, and the first moment of the frequency of the O─H stretch vibration derived from the FTIR spectra of the pure hydrated salts.

The structural molecular realities of H_2_O molecules can be monitored by FTIR spectroscopy. Figure 3a shows the FTIR spectra of the hydrated salts plotted against the position of the (OER onset‐) tangents derived from Figure 2. It can be seen that with an increasing redshift of the O─H stretch vibration band (from Co(NO_3_)_3_ · 6 H_2_O to Fe(NO_3_)_3_·9 H_2_O) the OER onset potential decreases. We obtained a good correlation between the position of the O─H stretch vibration as defined as the first momentum of the frequency, derived from FTIR spectroscopic studies of the hydrated salts, and the onset of the OER potential, derived from CV measurements using the tangent method (Figure 3b). No voltage drop correction (IR correction) was applied to the CV data shown in Figure 2. Voltage drop correction based on EIS measurements (Figures S20a–g) did not significantly change the results, the CVs of which are shown in Figures S30a–g and the IR‐corrected correlation is shown in Figure S31.

In addition to the tangent method, we calculated the onset of OER using the first derivative method, i.e., a threshold value of 0.5  and 1.0 mA/cm^2^ V for the first derivative of the CV curve was used to define the onset potential (Figure S32a, S32b). Again, a good correlation was obtained when the onset of OER was plotted against the first momentum of the frequency.

**Figure 4:**
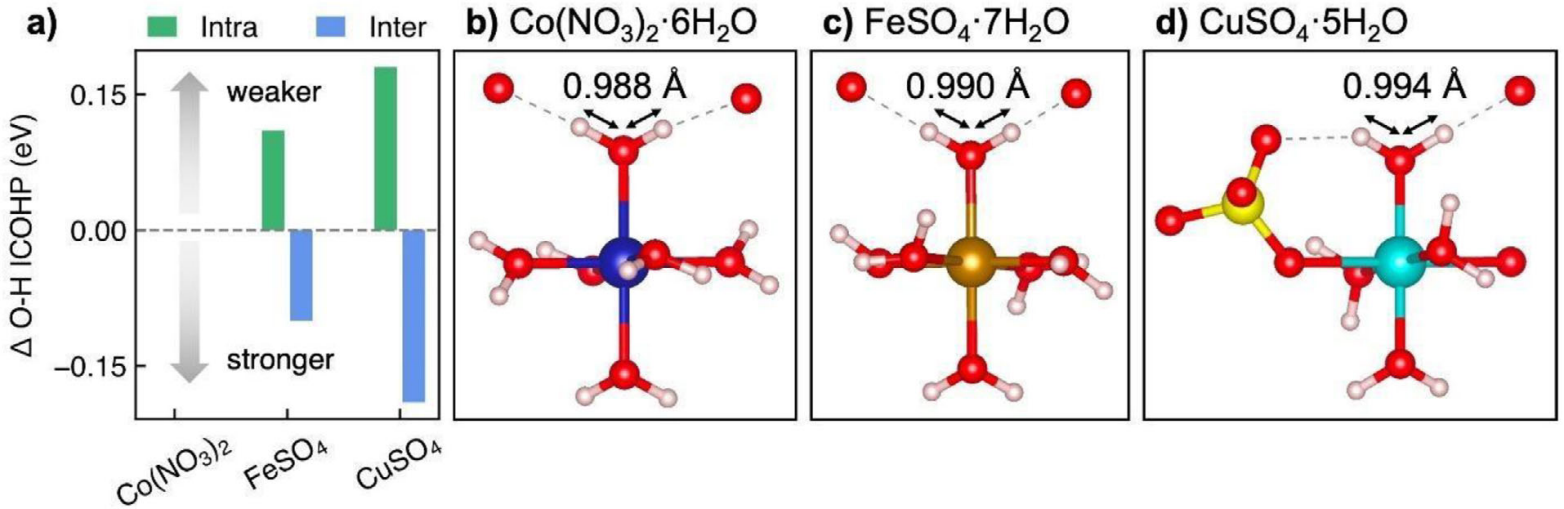
The bond strength of crystallised salts is manifest through the bond length. a) Integrated crystal orbital hamilton population (ICOHP) of intra‐ and inter‐molecular O─H bonds relative to Co(NO_3_)_2_·6. A more negative ICOHP indicates strong bonds. b–d) The structural‐molecular realities of crystallised water, based on density functional theory relaxations of b) Co(NO_3_)_2_·6 H_2_O, c) FeSO_4_·7 H_2_O, and d) CuSO_4_·5 H_2_O, respectively.

To investigate the atomistic origin of this trend, we performed density functional theory calculations (DFT) on a subset of the hydrated salts (Figure 4a–d). Through analysis of the optimised geometries, we observed that the O─H bond lengths increase monotonically from Co(NO_3_)_2_·6 H_2_O (0.988 Å) to FeSO_4_·7 H_2_O (0.990 Å) and CuSO_4_·5 H_2_O (0.994 Å). This trend directly correlates with the shift in the first moment of the IR frequencies and the onset of the OER potential observed experimentally, as illustrated in Figure 3b. In liquid water, the intramolecular O─H distance was determined to be 0.960 Å.^[^ ^33^ ^]^ To provide further insight into the chemical bonding underpinning this behaviour, we calculated the Integrated crystal orbital hamilton population (ICOHP). This quantity provides an estimate of the bond strength between two sites in a crystal structure, with a more negative number indicating great bonding character (stronger bonds). Our calculations reveal that the average intramolecular O─H bond strength decreases down the series from Co(NO_3_)_2_·6 H_2_O (−8.29 eV) to FeSO_4_·7 H_2_O (−8.18 eV) to CuSO_4_·5 H_2_O (−8.11 eV). This is accompanied by an increase in the intermolecular O─H bond strength from Co(NO_3_)_2_·6 H_2_O (−0.87 eV) to FeSO_4_·7 H_2_O (−0.97 eV) and CuSO_4_·5 H_2_O (−1.06 eV). These combined findings further reinforce our suggestion of intramolecular bond manipulation as the origin of the phenomena observed in experiments.

### Electrolysis of Hydrated Salt/DMSO Suspensions Carried Out in an Electrolysis Cell‐ A Proof of Concept

**Figure 5:**
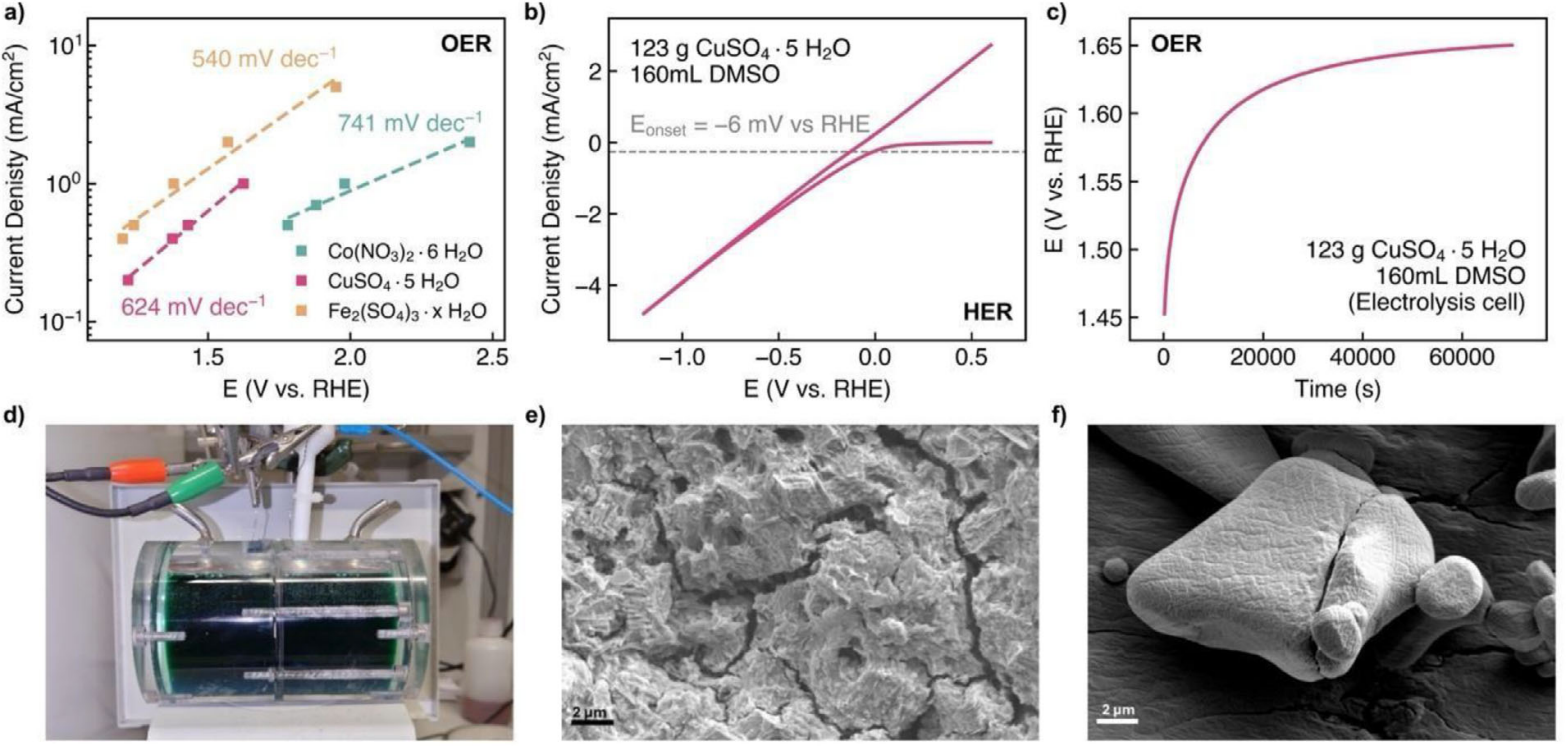
Results of electrochemical experiments carried out in an electrolysis cell a‐d) and SEM investigations e, f). The total volume of the electrolyte was about 220 mL. Approximately 120 g of each salt was added to 140–160 mL of DMSO. Electrode area of Pt WE: 1 cm2; Pt CE: 12 cm^2^ geometric area. a) Plot of Tafel slopes derived from CP scans (Figures S34, S35, S36) performed with hydrated salt/DMSO mixtures. b) CV plot performed with a CuSO_4_ ·5 H_2_O/DMSO suspension between 0.6 and −1.2 V versus RHE; scan rate: 10 mV/s. c) CP plot derived from a CuSO_4_ ·5 H_2_O/DMSO suspension at a current density of 1 mA/cm^2^. d) Photo of the electrolysis cell filled with CuSO_4_·5H_2_O/DMSO suspension. e) SEM image of hydrated CuSO_4_ used in an electrolysis cell for 167 h at a current density of 20 mA/cm^2^. f) SEM image of unused CuSO_4_·5H_2_O. The acceleration voltage was adjusted to 5 kV e) and 3 kV f). The SEM images were taken with a secondary electron detector.

As a proof of concept for full water electrolysis CV and galvanostatic (CP) measurements were carried out in a home‐built water electrolysis cell (Figures 5a–d, S33) equipped with a Nafion212 membrane that allows separation of the two compartments. DMSO suspensions of Co(NO_3_)_3_·6 H_2_O (Figures S34 a–e) CuSO_4_·5H_2_O (Figures S35, a–e) and Fe_2_(SO_4_)_3_·x H_2_O (Figures S36, a–f) were subjected to CP experiments. Tafel plots based on these galvanostatic measurements (Figure 5a) with Tafel slopes of 741 mV/dec (Co(NO_3_)_2_·6 H_2_O), 624 mV/dec (CuSO_4_·5 H_2_O) and 540 mV/dec (Fe_2_(SO_4_)_3_ x H_2_O) confirm what we learned from the CV tests, namely that Fe_2_(SO_4_)_3_ x H_2_O has the highest activity in supporting the oxygen evolution reaction, followed by CuSO_4_ 5 H_2_O and Co(NO_3_)_2_·6 H_2_O. It should be noted that the saturated suspensions based on organic solvents, with high viscosity and relatively low conductivity, cannot be compared with stirred aqueous electrolytes, e.g. in terms of mass transport phenomena. A CV curve, which typically occurs from the onset of the hydrogen evolution reaction, was obtained showing an onset potential of −6 mV versus RHE (Figure 5b). For the HER part onset of HER is defined as the point at which the current density falls below a current density of −250 µA/cm^2^. Long‐term CP tests have shown sufficient stability at both positive and negative potentials (Figures 5c, S37).

We determined the faradaic efficiency of the oxygen evolving reaction (see Methods in the Supporting Information Figure S38). Using an electrolyte that consisted of a Fe(NO_3_)_3_·9 H_2_O/DMSO suspension the faradaic efficiency turned out to be 88.4% for the anodic half‐cell reaction at a current density of 300 mA/cm2 (Figure S38). The mass loss of the electrolyte was determined to further confirm the successful decomposition of the crystallised water. We performed a chronopotentiometry measurement with 20 g CuSO_4_·5 H_2_O (80.1 mmol) in 23.5 mL DMSO for 167 h at *j* = 20 mA/cm^2^ and determined the mass deficit due to water splitting. The corrected mass deficit was 0.832 g, which is close to the value expected from a calculation using Faraday's law (1.13 g, see Supporting Information for details). Thus, around 11.5% (46.2 mmol) of the water present in the salt (400.5 mmol) has been split through 167 h of CP‐based water electrolysis. Using a smaller quantity of CuSO_4_·5H_2_O led to the near‐complete decolourisation of a dimethyl sulfoxide (DMSO) suspension during electrolysis (see Figure S39).

The loss of crystallised water is expected to change the morphology and surface texture of the salt particles which indeed was obtained. We therefore examined CuSO_4_·5 H_2_O by electron microscopy before and after subjecting it to water electrolysis for 167 h. For comparison, we examined CuSO_4_·5 H_2_O stirred in DMSO for 167 h. CuSO_4_·5 H_2_O that was subjected to electrolysis consists of particles with high surface roughness exhibiting holes and cracks (Figure 5e) whereas unused CuSO_4_ 5 H_2_O consists of rounded, compact particles with a closed surface (Figure 5f). At first glance, the appearance of rounded particles appears unusual for an inorganic salt but has been found in other SEM studies of CuSO_4_·5 H_2_O.^[^ ^34^ ^]^ CuSO_4_·5 H_2_O that was stirred in DMSO shows a fissured surface (Figure S40) compared to unused CuSO4·5 H2O, but not the cracks and holes visible in Figure 5e.

### Electrolysis of Aqueous Salt Solutions – Demonstration of the Feasibility of the Approach from a More Practical Perspective

We performed full water electrolysis in an electrolysis cell (Figure S41) using an anion exchange membrane Fumasep FAP‐450 from Fumatech and Pt as working‐, and counter electrode (Supporting Infomation). Electrolysis enabling galvanostatic conditions (chronopotentiometry tests) at current densities of 100‐, 150‐, 200‐, 225‐, 300‐, and 375 mA/cm^2^ (Figures S42–S47) were carried out, all of which using 110 mL of 3 M H_2_SO_4_ as the catholyte. The anolyte consisted of 110 mL of 0.125 M H_2_SO_4_ (i), 15 g of Fe_2_(SO_4_)_3_·x H_2_O (359 mmol) dissolved in 100 mL of 0.125 M H_2_SO_4_ (ii), 25.5 g of Na_2_SO_4_ (180 mmol) dissolved in 100 mL of 0.125 M H_2_SO_4_ (iii). A direct comparison of the results obtained using anolytes i) (Figures S42, S45) and ii) (Figures S43, S46) allows the usefulness of using aqueous salt solutions for water electrolysis to be assessed. However, in order to exclude the possibility that any improvement in the efficiency of water electrolysis obtained by comparing the results obtained with anolytes i and ii is simply due to a higher ionic strength of ii) (I = 5.76 mol/L) compared with i) (0.38 mol/L), we have also tested a solution of Na_2_SO_4_ in H_2_SO_4_ (iii; I = 5.76 mol/L) in water electrolysis experiments (Figures S44, S47). A look at the water electrolysis efficiencies (Table 1, S2) shows that water electrolysis with 3 M H_2_SO_4_ as catholyte and a solution of Fe_2_(SO_4_)_3_ in sulphuric acid as anolyte (ii) gave the highest efficiency with a total cell voltage of 2.42 V (at *j* = 300 mA/cm^2^; Figure S46). In particular, the cell voltage was 260 mV lower than in corresponding measurements carried out with pure 0.125 M H_2_SO_4_ electrolyte (Figure S45) and even 320 mV lower when using a Na_2_SO_4_/H_2_SO_4_ electrolyte (Figure S47) with the same ionic strength. More importantly, the significant reduction in cell voltage obtained for the different anolytes is accompanied by a significant reduction in the OER overpotential (*η*300 = 100 mV (ii; Figure S43); *η*300 = 291 mV (i; Figure S41); *η*300 = 311 mV (iii; Figure S44)) suggesting that the reduced cell voltage basically originates from the more efficient anodic half‐cell reaction.

**Table 1:** Results from water electrolysis tests carried out in an electrolysis cell (Figure S41) upon using 3 M H_2_SO_4_ as the catholyte and different anolytes (column I); cell voltage at *j* = 300 mA cm^−1^ (column II), OER overpotential at *j* = 300 mA cm^−1^ (column III), respectively. The values shown in columns two and three represent the arithmetic mean of cell voltages and OER overpotentials derived from 2700 s chronopotentiometry scans (Figures S42–S47). More detailed results can be found in Table S2 (Supporting Information).

Anolyte	Cell voltage [V]	OER η300 [mV]
i (0.125 M H_2_SO_4_)	2.68	291
ii (H_2_SO_4_/Fe_2_(SO_4_)_3_ ·H_2_O)	2.42	100
iii (H_2_SO_4_ /Na_2_SO_4_)	2.74	311

We have checked the Faraday efficiency for both half‐cell reactions in aqueous solutions during a chronopotentiometry measurement at I = 300 mA using our Fe_2_(SO_4_)_3_‐modified anolyte ii, (see Methods in the Supporting Information Figure S41) by collecting both gases. As expected, full charge to oxygen and charge to hydrogen conversion was determined with faradaic efficiencies of 99.5% (HER) and 99.3% (OER) (Figures S48 and S49).

In addition to determining the Faraday efficiency by collecting the gas produced, we checked the Faraday efficiency of the oxygen evolution reaction in anolyte ii by measuring the amount of dissolved oxygen using an O_2_ detector (Supporting Information). An important feature of this approach is that it measures the rate of oxygen evolution in a solution where the oxygen concentration is well below the saturation concentration (Supporting Information). The aforementioned Faraday efficiency of 99.3% (OER) was very well confirmed using this fluorescence quenching method (arithmetic mean:99.5%) see Figure S50–S52 (Supporting Information).

## Conclusions

In contrast to liquid water, the structural‐molecular realities of water molecules embedded in hydrated salts vary little and are under exceptionally strong influence, making them ideal candidates for the water splitting reaction. The ideal exploitation of this strategy requires that only embedded molecules are subjected to water electrolysis and that no “loose” water is added. In this work, we have succeeded in decomposing crystallised water for the first time. Water electrolysis was obtained from toluene/CuSO_4_·5 H_2_O and toluene/Al_2_(SO_4_)_3_·18 H_2_O slurries using a Pt/Pt electrode pair. By replacing toluene with DMSO, successful water electrolysis, as deduced from electrochemical measurements under quasi‐stationary (CV) and galvanostatic (CP) conditions, was confirmed for a variety of suspensions of hydrated salts (CuSO_4_, Co(NO_3_)_2_, FeSO_4_, Fe_2_(SO_4_)_3_, Fe(NO_3_)_3_, and Ni(SO_4_)). In addition, the frequency of the O─ H stretch vibration obtained from simple FTIR spectroscopic analysis of the solids (hydrated salts), makes low‐cost and readily available vibrational spectroscopy an ideal tool for predicting the activity of the water embedded in the hydrated salt towards water electrolysis. Our experimental findings are rationalised through comprehensive spin‐polarised first‐principles calculations that further point to the causal relationship between the position of the O─H stretch vibration and the potential at which oxygen evolution starts. Our work provides a framework for the manipulation of the bonds in crystallised H_2_O and a clear strategy for modulating the classical parameters of water electrolysis, enabling low overpotentials for both half‐cell reactions. We have also shown that the intramolecular bonding of water molecules in aqueous solutions can also be favourably manipulated by the use of dissolved salt, demonstrating the feasibility of the approach from a more practical perspective. We believe that this strategy should be seen as a new and reasonable alternative to existing and dominant approaches, such as the optimisation of electrode materials, which aim to make water electrolysis more efficient.

## Conflict of Interests

The authors declare no conflict of interest.

## Supporting information



Supporting Information

Supporting Information

Supporting Information

Supporting Information

## Data Availability

The data that support the findings of this study are available from the corresponding author upon reasonable request.
