# Supplementary material for: Water‐Splitting “Without Water”: Splitting of the Crystallised Water of Hydrated Salts
Source: Angew Chem Int Ed Engl. 2025 Dec 6;65(4):e20018. doi: 10.1002/anie.202520018 (PMC12828478; doi:10.1002/anie.202520018)
Supplement: Supplementary file 1 — Supporting Information [file ANIE-65-e20018-s002.pdf]

Supporting Information

WATER-SPLITTING "WITHOUT WATER": SPLITTING OF THE CRYSTALLISED WATER OF HYDRATED SALTS

Klara Rüwe[a], Shirui Wang[b], Tom Bookholt[a], Julia Brune[a], Hannelore Schmidt[c], Marius Behnecke[c], Svea Petersen[c], Claudia Hess[d], Alex M. Ganose[*b], and Helmut Schäfer[* a]

[a] *Department of Biology and Chemistry, The Electrochemical Energy and Catalysis Group, University of Osnabrück; Barbarastrasse 7, 49076 Osnabrück, Germany*
E-mail: helmut.schaefer@uos.de

[b] *Department of Chemistry, Molecular Sciences Research Hub, White City Campus, Imperial College London, Wood Lane, London W12 0BZ, UK*
E-mail: a.ganose@imperial.ac.uk

[c] *Faculty of Engineering and Computer Science, Laboratory for Organic Chemistry and Polymer Chemistry, University of Applied Sciences Osnabrück, P.O. Box 1940, 49009 Osnabrück, Germany*

[d] *Department of Biology and Chemistry, Physical Chemistry, University of Osnabrück; Barbarastrasse 7, 49076 Osnabrück, Germany*

Methods

Quantum Mechanical Calculations

All calculations in this work were performed using density functional theory (DFT) as implemented in the Vienna *Ab initio* Simulation Package (VASP)[1, 2, 3, 4]. VASP is a plane-wave code that employs the projector-augmented-wave (PAW) method to describe interactions between core and valence electrons [5]. To address the limitations of standard DFT in dealing with strongly correlated electron systems, we incorporated Hubbard U parameters of 3.32 eV for Co, 5.30 eV for Fe, and 5.17 eV for Cu in our DFT+U calculations. This approach partially corrects the self-interaction errors inherent in standard DFT, which often lead to the over-delocalisation of *d* electrons in transition metal compounds[6]. Spin-polarized calculations are performed for all three hydrated salts since the elements cobalt, iron, and copper in these compounds are in high-spin configurations (d^7 for Co, d^6 for Fe, and d^9 for Cu) with unpaired electrons leading to a more precise depiction of their electronic structures. The plane-wave energy cutoff and k-point mesh were converged to 5 and 1 eV per atom, respectively. Table S1 gives the converged cutoff and k-point meshes for the materials considered. Geometry optimisations were carried out using the Perdew-Burke-Ernzerhof (PBE) exchange-correlation functional [7] with the DFT-D3 dispersion correction[8] which has been shown to accurately predict the geometric properties of these hydrated salts. The theoretical lattice parameters and those determined from experiments differed by an average of 0.7%, highlighting the appropriate functional choice. To obtain estimates of the O-H bond strength, we performed Crystal Orbital Hamilton Population (COHP) analysis using the LOBSTER program[9]. COHP results were post-processed using the LobsterPy package[10].

Hydrated salts	Plane wave cut-off / eV	*k*-point mesh
$Co(NO_3)_2 \cdot 6H_2O$ [11]	600	3 × 3 × 1
$FeSO_4 \cdot 7H_2O$ [12]	600	1 × 2 × 1
$CuSO_4 \cdot 5H_2O$ [13]	600	2 × 2 × 2

Table S1: The converged plane wave energy cut-offs and *k*-point meshes used in this work.

The FTIR measurements were performed using a Bruker Vertex 70 FTIR spectrometer equipped with an ATR unit. The IR spectra were recorded at 2 cm^{-1} resolution in a spectral range from 400 to 4000 cm^{-1}. The computing of the first momentum of the frequency: Two location parameters were computed by the maximum of the transmittance f and by its first normalized moment ∫**Ω**xf(x)dx/f**Ω**f(x)dx where it was restricted to the field of view Ω = [2400,3800].

Electron microscopy

SEM images of $Cu(SO_4)_2 \cdot 5\ H_2O$ before and after long-term use in DMSO-based suspensions as electrolyte for water electrolysis were taken together with unused $Cu(SO_4)_2 \cdot 5\ H_2O$ using a Zeiss Auriga scanning electron microscope. The acceleration voltage was set to 3-5 kV and the SEM images were acquired using a secondary electron detector.

Sample preparation: At the end of the long-term electrolysis (167 h) carried out with 20 g $CuSO_4 \cdot 5\ H_2O$ in 23.5 mL DMSO at a current density of 20 mA/cm^2, the electrolyte was centrifuged and the precipitate dried for 72 h at 358.15 K in a vacuum furnace at a pressure of about 5 mbar. The control sample (20 g $CuSO_4 \cdot 5\ H_2O$ stirred for 167 h in 23.5 mL DMSO) was obtained by centrifuging the precipitate and drying the residue for 72 h at 358.15 K in a vacuum furnace at a pressure of about 5 mbar.

The elemental analysis of the surface of the Pt electrode after being used as the working electrode for recording 15 CV scans with 20 g $CuSO_4 \cdot 5\ H_2O$ (80.1 mmol in 22 mL DMSO, 17.38 g (59.7 mmol) of $Co(NO_3)_2 \cdot 6\ H_2O$ in 20 mL of DMSO, respectively was carried out using using a Zeiss Auriga scanning electron microscope equipped with an Oxford energy dispersive X-ray spectroscopy (EDS) instrument. The acceleration voltage was set at 5 kV and the SEM images were acquired using a secondary electron detector at a working distance of 5 mm. Actec software was used for image analysis.

Electrochemical Measurements

A three electrode set-up was used for all electrochemical measurements. The Pt WE was made of Pt wire (Evochem Advanced Materials GmbH, Offenbach am Main, Germany), 0.5 mm in diameter, 6.0 cm in length which corresponded to an electrode area of 1 cm^2. A Pt wire (Evochem Advanced Materials GmbH, Offenbach am Main,
Germany) electrode (3 ×4 cm geometric area) was exploited as counter electrode and a RHE (Hydroflex, Gaskatel Gesellschaft für Gassysteme durch Katalyse und Elektrochemie mbH, Kassel, Germany) was used as the reference electrode. In some cases, a silver-silver chloride (Ag/AgCl) electrode (Deutsche Metrohm GmbH & Co. KG; Kaninenberghöhe 8, 45136 Essen, Germany) with c = 3 mol L-1 KCl is used as a reference electrode. The distance between RE and WE was set at 3 mm and the distance between CE and RE was set at 4 mm. All electrochemical data were recorded digitally using either a Potentiostat Keithley Tektronix 2460 SourceMeter (Keithley Instruments GmbH, 82110

Germering, Germany), an Interface 1010 from Gamry Instruments (Warminster, PA 18 974, USA) connected to a personal computer or an PGStat 20 from Autolab (No voltage drop compensation was performed except for the measurements shown in Figure S30.

IR correction (voltage drop compensation)

Voltage drop compensation (IR correction) was applied to the CV data shown in Figure S30. The IR correction is based on a 30% correction using the electrolyte resistance as derived from EIS spectroscopic measurements (Figure S20). For example, in the case of DMSO/$FeSO_4 \cdot 7\ H_2O$, the potential on the abscissa of the CV was corrected by a resistance of 114 Ω (30% of Re=380 Ω). Correcting the CV by more than 30% resulted in an abnormal voltage-current dependence in some of the plots (increase in current density with decreasing potential).

Cyclic voltammetry experiments performed with organic solvent/salt mixtures.

Cyclic voltammetric measurements were carried out according to the following protocol: I) The counter electrode (Pt) and the working electrode (Pt) were cleaned by ultrasonication in HCl (10% by weight) for 15 min at room temperature. II) The electrodes were rinsed in distilled water for 2 minutes.

This procedure (steps I-II) was repeated after each CV experiment (consisting of three CV cycles) (*i.e.* after each measurement performed with a specific salt/DMSO or salt/toluene mixture, respectively, in order to minimise any change in the working electrode during the performance of a CV sweep). The equilibration time (at E=1.0 V vs. RHE) was set to 10 s. Except for the CVs performed with $Co(NO_3)_2 \cdot 6\ H_2O$/DMSO slurries, no pre-oxidation steps were applied. In the case of the $Co(NO_3)_2 \cdot 6\ H_2O$/DMSO slurry a pre-oxidation at 1.50 V vs. RHE was first applied for 700 s before starting the CV scan to eliminate the influence of Co(II)-Co(III) oxidation on the subsequent CV scan.

The scan rate was set to 10 mV/s. The third CV cycle is presented in the Figures, e.g. Figure 2.

Electronic Impedance spectroscopy (EIS)

Impedance spectroscopy of the samples was conducted using either DMSO, a slurry consisting of 34.5 g $CuSO_4 \cdot 5\ H_2O$ (216.16 mmol)/40 mL toluene; 20 g $CuSO_4 \cdot 5\ H_2O$ (80.1 mmol) / 20 mL DMSO, 17.38 g (59. 7 mmol) $Co(NO_3)_2 \cdot 6\ H_2O$/20 mL DMSO; 20 g (80.1 mmol) $CuSO_4 \cdot 5\ H_2O$/22 mL DMSO; 21.1 g (52. 2 mmol) $Fe(NO_3)_3 \cdot 9\ H_2O$/19 mL DMSO; 23.31 g (88.6 mmol) $NiSO_4 \cdot 6\ H_2O$/20 mL DMSO; 22.27 g (80. 1 mmol) $FeSO_4 \cdot 7\ H_2O$/40 mL DMSO and 22.27 g (53.3 mmol) $Fe_2(SO_4)_3 \cdot x\ H_2O$/ 40 mL DMSO within a frequency range 0.1-50469 Hz with an Autolab PGStat 20 potentiostat, controlled by *FRA* Windows software
(Frequency Response Analysis for Windows version *4.9.007*). To ensure accurate results more five measurements were made for each sample at defined potential. The reported results in this paper are average ones of the five derived from each sample. Electrode area of Pt WE: 4 cm^2; Pt CE: 3 cm^2 geometric area.

Electrolysis of hydrated salt/DMSO suspensions carried out in an electrolysis cell

A home-made electrolysis cell measuring 12.5 x 8.2 x 7.5 cm (length x width x height); inner diameter: 5 cm; volume: 220 cm^3 (Figure S33) was used. Approximately 120 g of each salt (122 g $Co(NO_3)_2 \cdot 6\ H_2O$, 123 g $CuSO_4 \cdot 5\ H_2O$, 124 g $Fe(SO_4)_3 \cdot x\ H_2O$) was added to 140-160 mL DMSO. The

electrolyte was then divided between the two half cells and the working, counter and reference electrodes were placed in the compartments (working electrode plus reference electrode in the anode compartment). Electrode area of Pt WE: 1 cm^2; Pt CE: 12 cm^2 geometric area. For the Faradaic efficiency measurements, the compartments were sealed with silicone sealant (Figure S38).

Tafel plots. Average voltage values for the Tafel plots were derived from 20000 second chronopotentiometry scans at current densities of 0.2, 0.5, 0.7, 1.0, 2.0 mA/cm^2 ($Co(NO_3)_2 \cdot 6\ H_2O$); 0.1, 0.2, 0.4, 0.5, 1.0 mA/cm^2 ($Cu(SO_4)_2 \cdot 5\ H_2O$); 0.2, 0.4, 0.5, 1.0, 2.0, 5.0 mA/cm^2 ($Fe_2(SO_4)_3 \cdot x\ H_2O$). The arrangement of RE, WE and CE for recording the chronopotentiometry plots was as described above (see Electrochemical Measurements section). The Pt electrodes were cleaned (steps I-II above) after each CP measurement.

Electrolysis of aqueous solutions in the electrolysis cell

A home-made electrolysis cell was used, see text above. Electrode area of Pt WE: 3 cm^2; Pt CE: 4 cm^2 geometric area (Figure S41). The catholyte was stirred while the anolyte was circulated with a small pump (Modell: GRL-14164 delivered by Botland. BOTLAND B. DERKACZ SP. K. Gola 25A, 63-640 Bralin, Poland). The distance between the counter electrode and the working electrode was set at 2 mm. The cathode compartment was filled with 110 mL of 3 M H_2SO_4 and the anode compartment with 110 mL of 0.125 M H_2SO_4 (i), 15 g of $Fe_2(SO_4)_3 \cdot x\ H_2O$ (0.36 mol/ L) dissolved in 100 mL of 0.125 M H_2SO_4 (ii), 25.5 g of Na_2SO_4 (180 mmol) dissolved in 100 mL of 0.125 M H_2SO_4 (iii), respectively. Cell voltage measurements were made during the measurement of chronopotentiometric scans using a multimeter with the ability to log cell voltage data (UNI-T UT 71C from UNI-TREND Technology, Dongguan City, Guangdong Province, China).

Determination of the Faradaic efficiency for the hydrogen evolving-, and oxygen evolving reaction in aqueous solutions by collecting both gases produced

A home-made electrolysis cell (Figure S33, 40) was used, see text above. We determined the faradaic efficiency by collecting the oxygen gas and hydrogen gas produced in the electrolysis cell during a chronopotentiometry test as described in our previous paper[14] at a current of 300 mA using a Pt WE (A=3 cm^2) and a Pt CE (A=2 cm^2) as well as a RHE as RE. U_{cell}=2.14 V. Separation of the cell compartments was ensured by using a Fumatech Fumasep FAP 450 anion exchange membrane to prevent reduction of Fe(III) to Fe(II) by reaction with the cathode. Briefly: The tubes of the anode and cathode chambers were connected with silicone hoses and the gas produced displaced the liquid column in an upright measuring cylinder. The catholyte (110 mL of 3 M H_2SO_4), was stirred while the anolyte (100 mL of 0.125 M H_2SO_4 + 15 g of $Fe_2(SO_4)_3 \cdot H_2O$) was circulated with a small pump (Modell: GRL-14164 delivered by Botland. BOTLAND B. DERKACZ SP. K. Gola 25A, 63-640 Bralin, Poland). The temperature was 293 K. The linear equation to calculate the amount of gas y (mL) produced as a function of the measurement time x (s) at 100% Faraday efficiency is as follows:

HER: $y=3.763 \cdot 10^{-2} \cdot x$

OER: $y=1.881 \cdot 10^{-2} \cdot x$

Figures S47, S48 show the straight lines that represents the quantities of gas at a Faraday efficiency of 100% together with the amount of gas gathered at the measurement time x

Determination of the Faradaic efficiency for the oxygen evolving reaction in aqueous solutions by the fluorescence quenching method

This method is limited to the determination of the Faradaic efficiency of the oxygen evolution reaction, as there is no suitable detector for the determination of hydrogen in solution.

The method was carried out in close accordance with the procedure described in our previous paper[15]. The basic setup is shown in Figure S41 (Pt WE (A=3 cm^2) and a Pt CE (A=2 cm^2). No reference electrode was used. Separation of the cell compartments was ensured by using a Fumatech Fumasep FAP 450 anion exchange membrane to prevent reduction of Fe(III) to Fe(II) by reaction with the cathode. The oxygen concentration in the electrolyte was measured with an optical dissolved oxygen (OD) sensor using the fluorescence quenching method (Multi 3420 IDS from WTW, Weilheim, Germany). Before starting the measurement, the electrolyte (catholyte: 110 mL of 3 M H_2SO_4, anolyte: 100 mL of 0.125 M H_2SO_4 + 15 g of $Fe_2(SO_4)_3 \cdot H_2O$) was added to both compartments and flushed with argon for 45 min at a constant flow rate of 0.7 cm^3/s until the dissolved oxygen reached 0.09 mg/L (measurement 1), 0.08 (measurement 2) and 0.11 (measurement 3), respectively. The total amount of anolyte was 110 mL. The anode compartment was completely sealed before chronopotentiometry was started. Dissolved oxygen values were recorded digitally using an instrument (WTW Multi 3420 IDS) connected to a personal computer. The power source was a Keithley 2401. The current was set at 50 mA. U_{cell}= 1.55 V.
The Faraday efficiency amounted to 99.5% (arithmetic mean) after 200 s of operation (Figure S49-S51)

The linear equation to calculate the oxygen content y (mg/L) reached in solution as a function of the measurement time x (s) at 100% Faraday efficiency is as follows:

OER: $y=3.768 \cdot 10^{-2} \cdot x + 0.09$ (measurement 1)

OER: $y=3.768 \cdot 10^{-2} \cdot x + 0.08$ (measurement 2)

OER: $y=3.768 \cdot 10^{-2} \cdot x + 0.11$ (measurement 3)

Figure S49-51 shows the straight line that represents the oxygen content (mg/L) at a Faraday efficiency of 100% together with the oxygen content (mg/L) determined at the measurement time x

Preparing the toluene/salt slurry

To 54 g (80.1 mmol) of $CuSO_4 \cdot 5\ H_2O$ (99%, Grüssing, Filsum, Germany), 40 mL of toluene (99%, Fisher Scientific, Japan) was added without stirring, and then the three electrodes were inserted into the slurry and the CV measurement started. Similarly, 40 mL of toluene was added to 34.5 g (216.16 mmol) of $CuSO_4$ (98%, Alfa Aesar, Kandel, Germany) and 40 mL of toluene was added to 26.69 g (40.05 mmol) of $Al_2(SO_4)_3 \cdot 18\ H_2O$ (99.9%, Acros Organics, New Jersey, USA).

Preparing the DMSO/salt suspensions

Care was taken to ensure that a suspension was produced in each case; the total volume of the suspensions was approximately 40 mL, *i.e.* 17.38 g (59. 7 mmol) of $Co(NO_3)_2 \cdot 6\ H_2O$ (Riedel de Haen, Seelze, Germany) was added to 20 mL of DMSO (Fisher Scientific, Bremen, Germany) with stirring; 20 g (80.1 mmol) of $CuSO_4 \cdot 5\ H_2O$ (99%, Grüssing, 26847 Filsum, Germany) was added to 22 mL of DMSO; 21.1 g (52. 2 mmol) $Fe(NO_3)_3 \cdot 9\ H_2O$ (Merck KGaA, Darmstadt, Germany) to 19 mL DMSO; 23.31 g (88.6 mmol) $NiSO_4 \cdot 6\ H_2O$ (Sigma-Aldrich, St. Louis, USA) to 20 mL DMSO; 22.27 g (80. 1 mmol) $FeSO_4 \cdot 7\ H_2O$ (99%, Sigma-Aldrich, St. Louis, USA) to 40 mL DMSO and 22.27 g (53.3 mmol) $Fe_2(SO_4)_3 \cdot x\ H_2O$ (98%, Carl Roth, Karlsruhe, Germany) to 40 mL DMSO.

Determination of the onset of oxygen evolution potential upon using the tangent method (applied to CV plots), *i.e.* the current density is back extrapolated to find the potential at which the extrapolation crosses the baseline (zero current density) (steps 1-4):

1. A potential range from 0.8 V vs. RHE to 2.5 V vs. RHE is shown on the abscissa. 2. An aspect ratio (ordinate to abscissa) of 1 to 1.35 has been set for all graphs. 3. The scaling is set so that a straight line from the point (0.8 V vs. RHE/ 0 mA/cm^2) to the point passing through the maximum current density (at reverse potential) makes an angle of 35° with the abscissa. 4. The potential at which a tangent to the CV curve with a slope of 45 degrees crosses the baseline (zero current density) is defined as the (onset of oxygen evolution) potential (Figures 2). In addition to this method, a threshold value of 0.5 mA/cm^2 V, 1.0 mA/cm^2 V, respectively for the first derivative of the CV curve was used to define the onset potential (Figure S32).

Determination of the onset of oxygen evolution potential based on IR corrected data

The abscissa starts at 1.0 V vs. RHE where the current density is 0 mA/cm^2. An aspect ratio (ordinate to abscissa) of 1:1.35 has been set for all graphs. 3. The scaling is set so that a straight line from the point (1.0 V vs. RHE/ 0 mA/cm^2) to the point passing through the maximum current density (at 2.30 V vs. RHE) makes an angle of 35° with the abscissa. The potential at which a tangent to the CV curve with a slope of 45 degrees crosses the baseline (zero current density) is defined as the (onset of oxygen evolution) potential (Figures S30).

Determination of the Faradaic efficiency for the oxygen evolving reaction in DMSO

We determined the faradaic efficiency by collecting the oxygen gas produced in the electrolysis cell (Figure S33) during a chronopotentiometry test as described in our previous paper[16] at a current density of 300 mA/cm^2 using a Pt WE (A=1 cm^2) and a Pt CE (A=2 cm^2) as well as a RHE as RE. The electrolysis cell was filled with a suspension consisting of 189.9 g $Fe(NO_3)_3\cdot 9H_2O$ + 171 mL DMSO. A Keithley Tektronix 2460 SourceMeter potentiostat was used as the current source. The current was set to 300 mA (j=300 mA/cm^2) ; U_{cell} =3.72 V). The temperature was 296.15K. At this temperature a production rate of 0.01887 mL/s is expected at 100% faradaic efficiency. After 420 s of operation (7 minutes), the total volume of oxygen produced was 7.0 mL, giving a faradaic efficiency of 88.4%.

Determination of the amount of water decomposed whilst long term electrolysis of DMSO/salt mixture

DMSO (23.5 mL) was added to 20 g $CuSO_4\cdot 5H_2O$ in a 50 mL electrolysis cell purged with dry Ar to prevent addition of water from the air. Electrolysis was started using a three-electrode configuration as above. The current was set to 20 mA (j=20 mA/cm2) and the running time to 167 h. The weight of the suspension + electrolysis cell (without stirring bar and electrodes) before and after long-term electrolysis was determined using a precision balance (Sartorius 1712). The loss of mass during electrolysis was 0.878 g (M1). In a control experiment, we again added 20 g $CuSO_4\cdot5H_2O$ to 23.5 g

DMSO in a 50 mL electrolysis cell and also determined the total mass (salt + DMSO + electrolysis cell). The suspension was stirred for 167 h and, at the end of the experiment, the mass deficit due to solvent loss based on purging with Ar was determined (M2). This mass deficit (M2=0.046 g) was subtracted from M1 to give the corrected mass deficit. The corrected mass deficit was 0.832 g, which is the amount of water decomposed during electrolysis. Based on 100% Faradaic efficiency, we would expect a mass loss of 1.13 g, calculated using Faraday's law:

6 $H_2O \rightarrow O_2 + 4\ H_3O^+$ ($4\ H_2O + 4\ H^+$) + 4 e^-, *i.e.* for 1 mol of H_2O 2 mol e^- are converted.

➡ 1 mol e^- ≙ 96486 As ≙ 0.5 mol H_2O ≙9.08 g

➡ 167 h ≙ 600000 s; Q=I · t=12000 As

12000 As ≙ 1.13 g (theoretical mass deficit)

Determination of the water content in toluene which was in contact with $CuSO_4 \cdot 5\ H_2O$ for 2 h and unused toluene upon Karl Fischer titration

Device: Baur KFM 3000

The titration cell is prepared by drying and filling with 100 ml Anolyt Coulomat AG and 5 ml Katholyt Coulomat C. The cell is conditioned for about 2 hours, i.e. titrated without water to a low drift value (approx. 1.9 µg/min) and checked with a water standard.

The sample material is withdrawn using a 1 ml syringe with cannula pre-conditioned with the sample and the total mass is determined on an analytical balance. Depending on the expected water content, a mass of between 40 and 700 mg, determined by differential weighing, is transferred to the titration cell via a septum. The drift at the beginning of the measurement was between 1.6 and 2.3 µg/min, depending on the expected water content, and the titration time was between 90 and 200 s. The water content is automatically calculated taking into account the sample mass and is given in ppm. Five measurements were made with $CuSO_4 \cdot 5\ H_2O$ and three with unused toluene. The arithmetic mean was 72.8 ppm ($CuSO_4 \cdot 5\ H_2O$) and 70.3 ppm (toluene) (**see results of the detailed tests below**).

Chemicals: Hydranal Coulomat AG Honeywell/Fluka, Cat. No. 34836 / Hydranal Coulomat CG Riedel de Haen Cat. No. 34840

Decolorization whilst electrolysis of $CuSO_4 \cdot 5\ H_2O$/DMSO suspension

It is worth noting the enormous hygroscopic nature of DMSO. 215 ml of DMSO was added to 6 g of $CuSO_4 \cdot 5H_2O$ (24 mmol) in a 250 ml electrolysis cell that was purged with dry Argon to prevent the addition of water from the air, while still allowing oxygen and hydrogen to leave the vessel. No membrane was used, as the aim of this experiment was not to analyse the splitting products, oxygen and hydrogen. Electrolysis was started using the above three-electrode configuration (Pt as WE and CE). The electrode area of both electrodes was 3 cm². The current was set to 60 mA (j = 20 mA/cm²). Almost complete decolourisation was obtained after around 145 hours of operation (see Figure S39, panels a–f). According to the 96485 As equivalent to 0.5 mol of decomposed water, a time of 107.34 hours was theoretically expected. It should be noted that, despite our best efforts to exclude water from the ambient air, we cannot be sure that we succeeded completely.

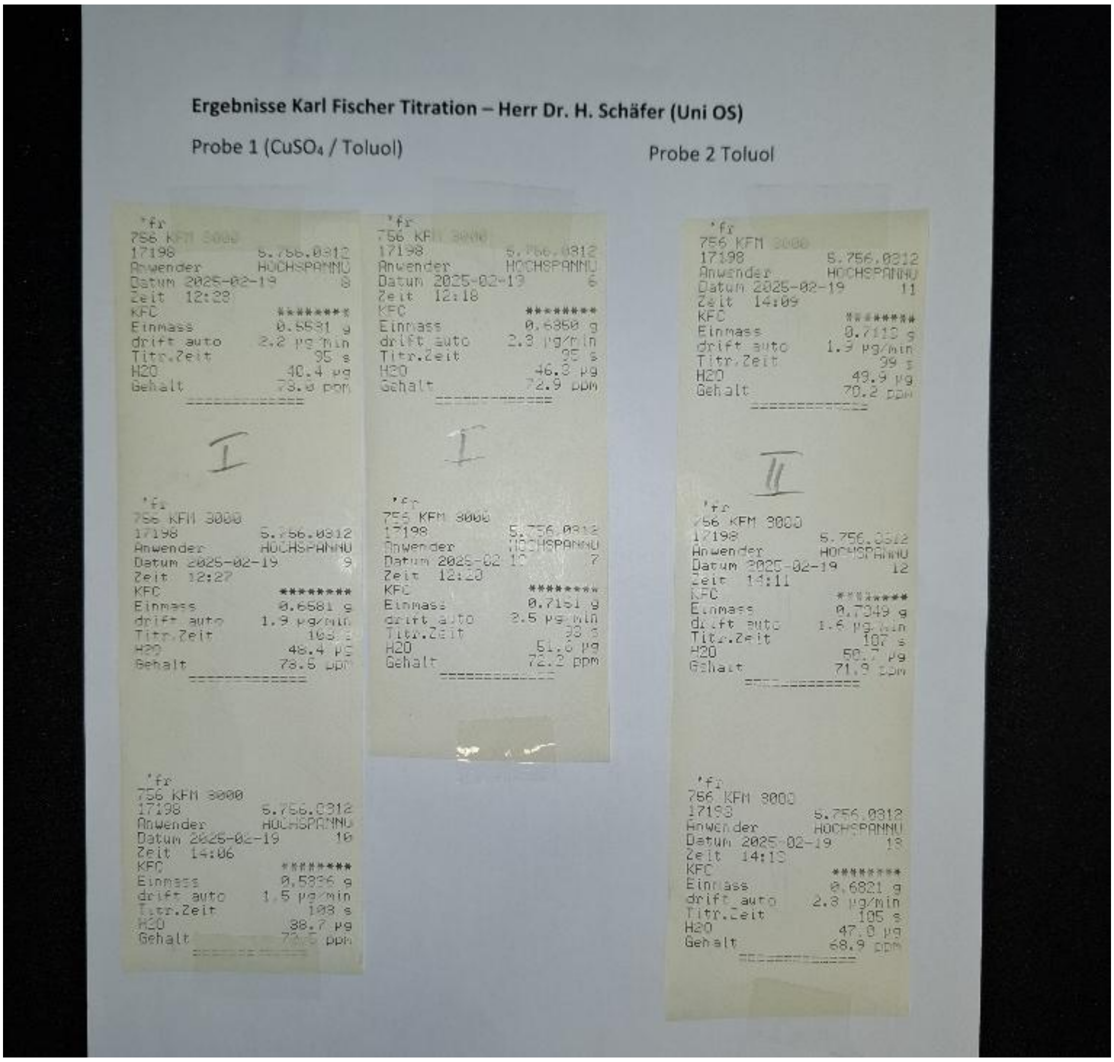

Electrolysis of a mm sized $CuSO_4 \cdot 5\ H_2O$ crystal under toluene

An almost saturated solution of $CuSO_4 \cdot 5\ H_2O$ in distilled water was obtained by adding 30 g of $CuSO_4 \cdot 5\ H_2O$ to 100 mL of distilled water. The solution was placed in a refrigerator to cool to 5 °C. After a few days, mm sized crystals can be taken directly from the solution. A petri dish of 9 cm diameter was filled with toluene and a $CuSO_4 \cdot 5\ H_2O$ crystal (9x6x5 mm) which was dried with a tissue and washed with toluene was placed in it (completely covered with toluene). The crystal was contacted under high pressure by precision spring contacts with hard gold plated tips and connected to a DC power source (Voltcraft VLP-2403 from Conrad Electronic SE; 92240 Hirschau, Germany). The voltage was set at 8V.

GC-MS headspace-based analysis of the gas formed upon electrolysis of a $CuSO_4 \cdot 5\ H_2O$ crystal

A 20 mm diameter headspace cap was placed over the crystal (see Figure S11) so that the bottom edge was just dimmed in toluene and the headspace was purged with helium by filling the headspace with helium gas using a 10 mL syringe. A sample of the headspace gas was taken with a GC syringe and injected into the GS-MS system. The crystal was connected to an electric current (see description above) and the measurement was repeated for 200 s during gas release.

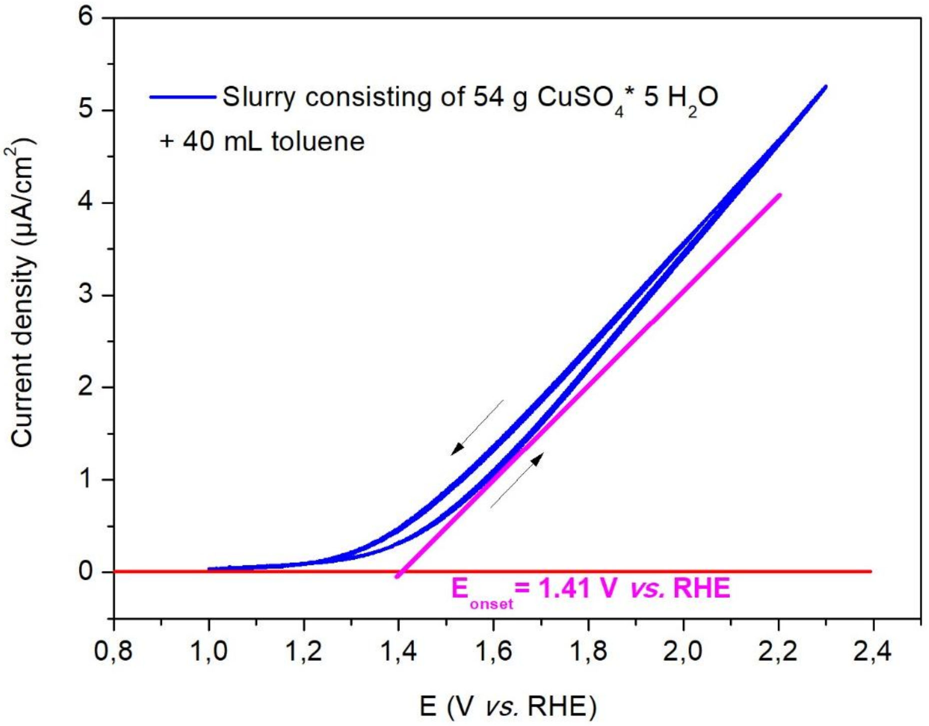

Figure S1. Cyclic voltammogram of the $CuSO_4{\cdot}5\ H_2O$ /toluene slurry. Onset potential of OER: 1.41 V *vs.* RHE as determined by the tangent method. Scan rate: 20 mV/s. WE= Pt (A=4 cm^2); CE= Pt (A=3 cm^2).

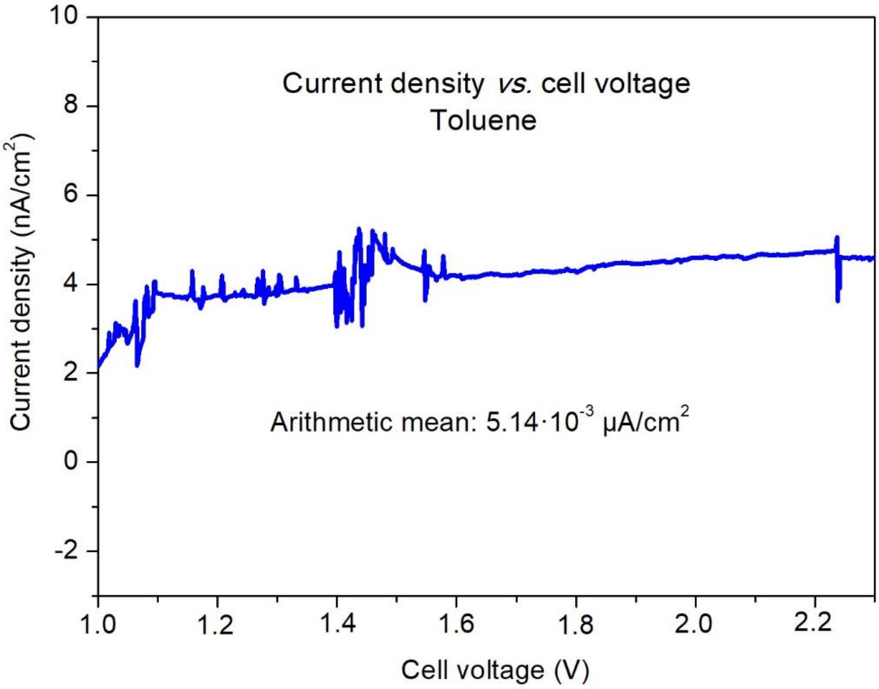

Figure S2.Current density vs. cell voltage plot of toluene. Electrode material: WE= Pt (A=4 cm^2); CE= Pt (A=3 cm^2). Current density was checked during the measurement of amperometry scans using a Keithley 2635B SourceMeter.

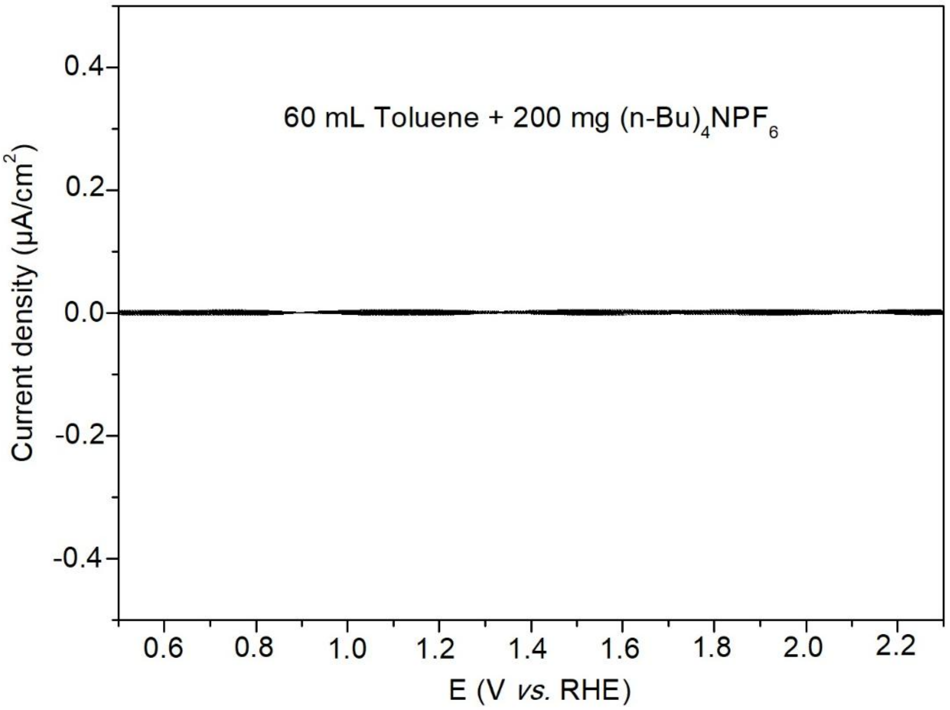

Figure S3. Cyclic voltammogram of toluene/(n-Bu)$_4$NPF$_6$ solution using a RHE as RE. WE= Pt (A=3 cm^2); CE= Pt (A=4 cm^2).

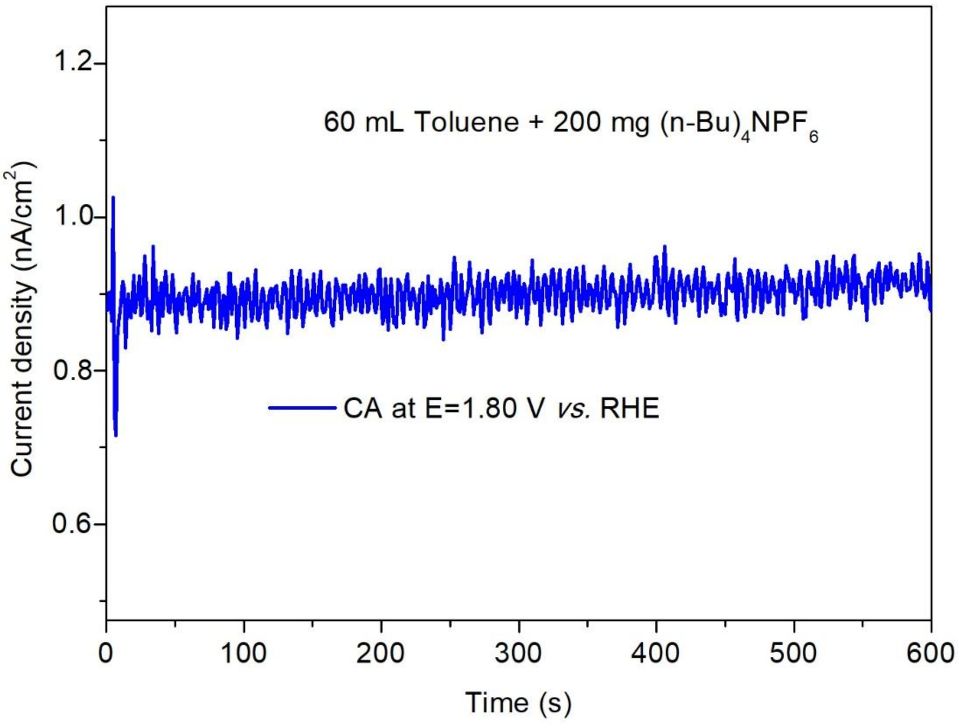

Figure S4. Chronoamperometry experiment carried out with toluene/(n-Bu)$_4$NPF$_6$ solution using a RHE as RE (E= 1.80 V *vs.* RHE). WE= Pt (A=3 cm^2); CE= Pt (A=4 cm^2).

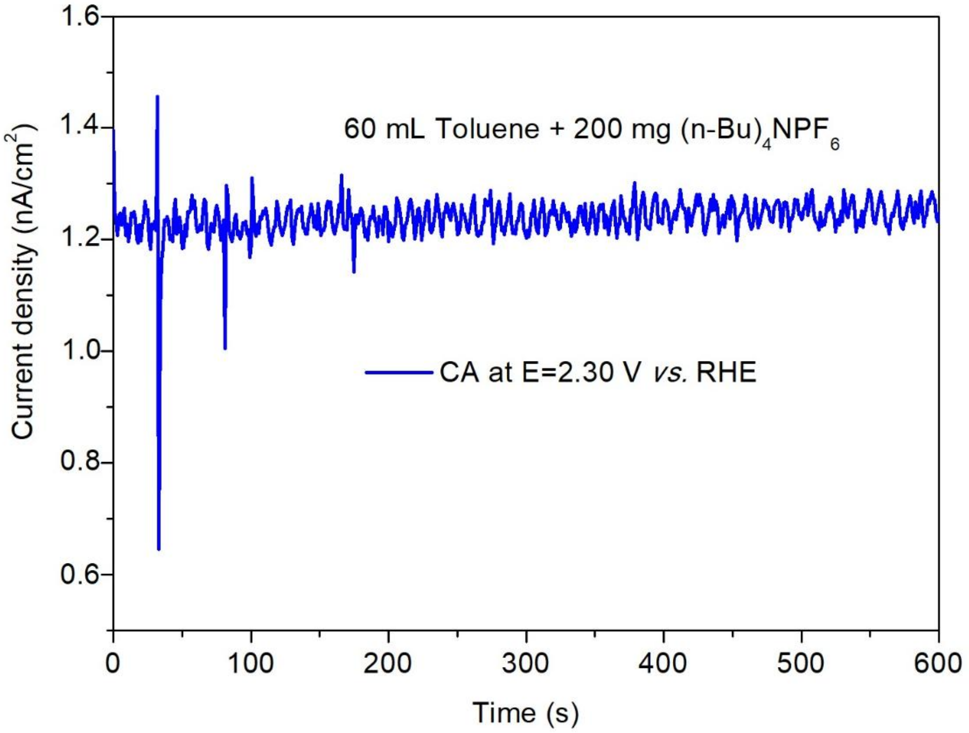

Figure S5. Chronoamperometry experiment carried out with toluene/(n-Bu)$_4$NPF$_6$ solution using a RHE as RE (E= 2.30 V *vs.* RHE). WE= Pt (A=3 cm^2); CE= Pt (A=4 cm^2).

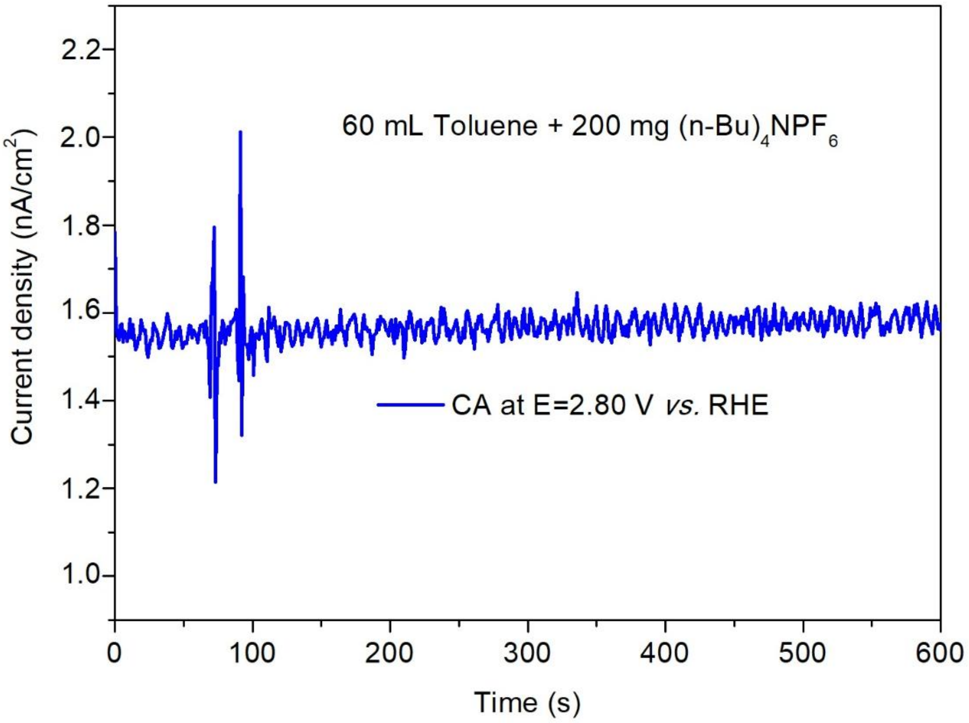

Figure S6. Chronoamperometry experiment carried out with toluene/(n-Bu)$_4$NPF$_6$ solution using a RHE as RE (E= 2.80 V *vs.* RHE). WE= Pt (A=3 cm^2); CE= Pt (A=4 cm^2).

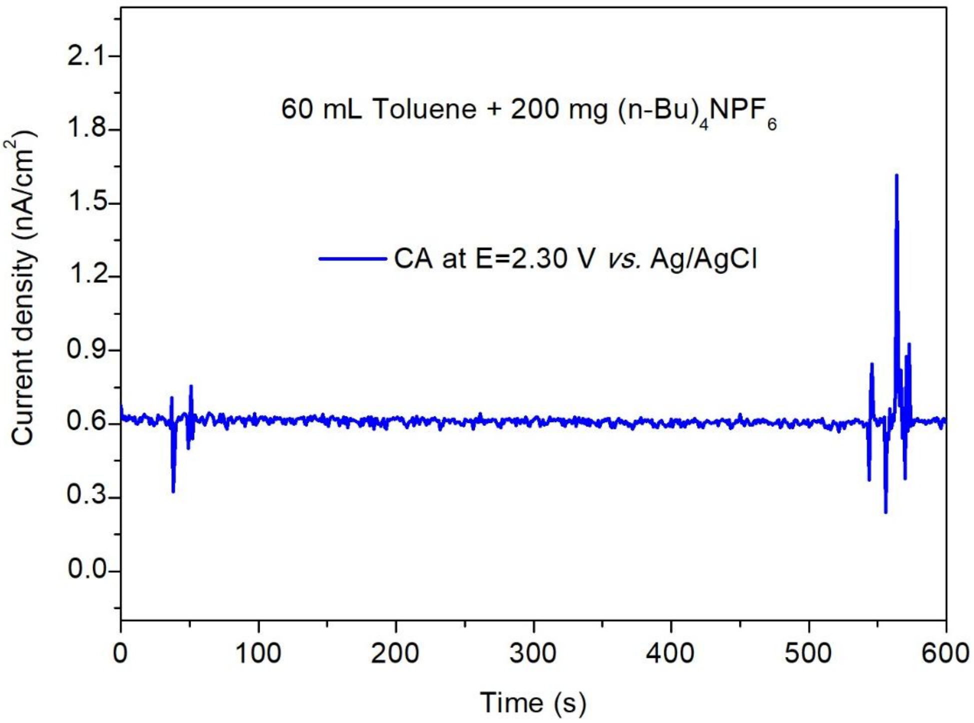

Figure S7. Chronoamperometry experiment carried out with toluene/(n-Bu)$_4$NPF$_6$ solution using a RHE as RE (E= 2.83 V *vs.* Ag/AgCl). WE= Pt (A=3 cm^2); CE= Pt (A=4 cm^2).

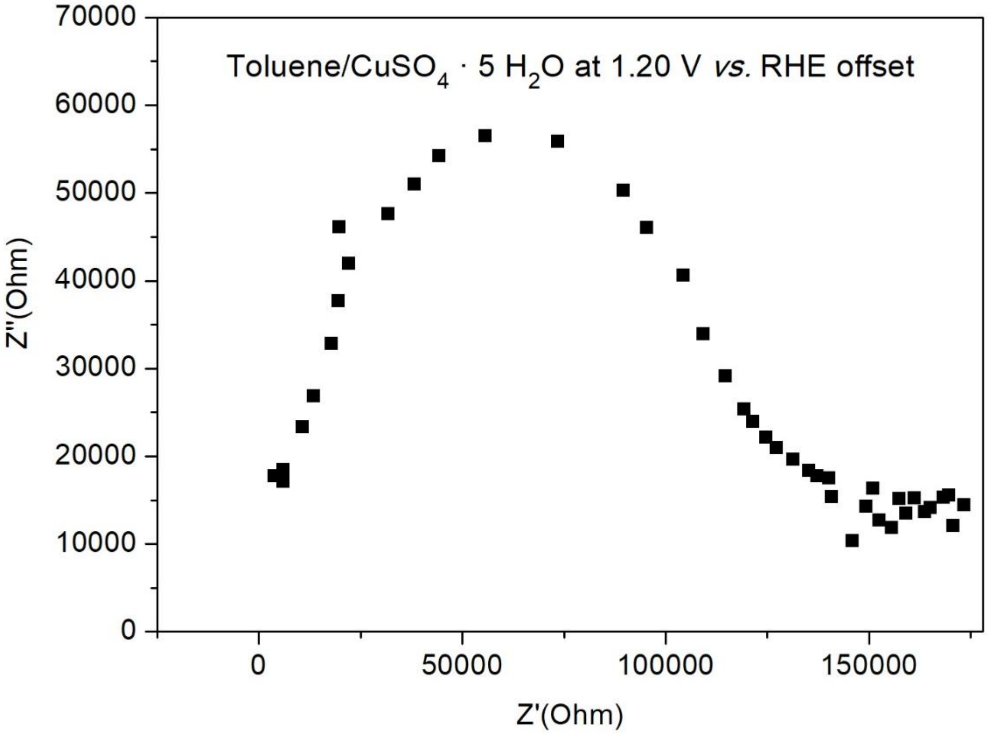

Figure S8. Nyquist plot of the frequency response analysis of a toluene/ $CuSO_4 \cdot 5\ H_2O$ slurry at an offset potential of 1.2 V vs. RHE. WE= Pt (A=3 cm^2); CE= Pt (A=4 cm^2).

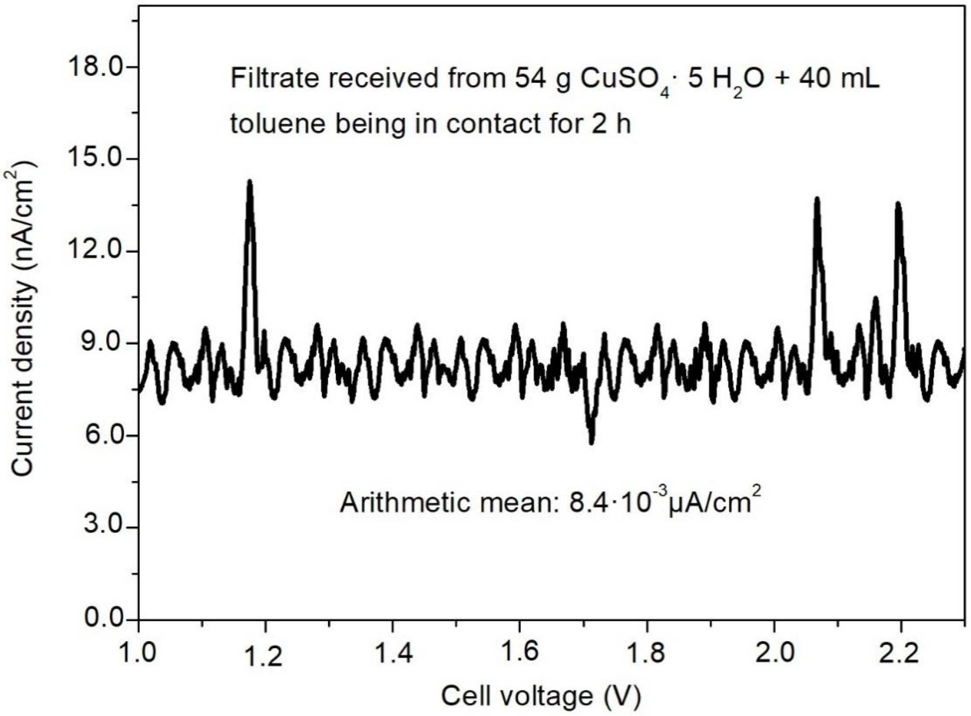

Figure S9. Current density vs. cell voltage from a solution obtained after filtration of 54 g $CuSO_4 \cdot 5\ H_2O$ + 40 mL toluene in contact for 2 h. Due to the very low conductivity, no CV could be recorded because the potentiostat reaches its voltage limit (the potential of the WE cannot be adjusted to the values normally indicated on the x-axis). Current density was checked during the measurement of amperometry scans using a Keithley 2635B SourceMeter. WE= Pt (A=4 cm^2); CE= Pt (A=3 cm^2).

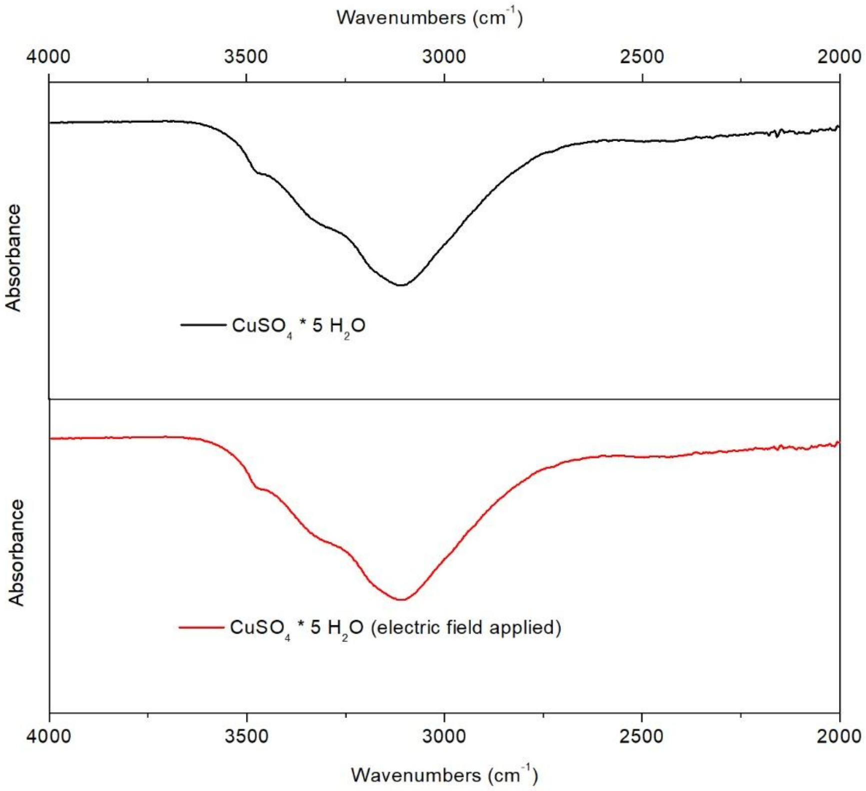

Figure S10. FTIR spectroscopic investigation of a $CuSO_4\ 5 \cdot H_2O$ crystal, 9x6x5 mm in size without and with (E= 1.4 Vmm^{-1}) electric current.

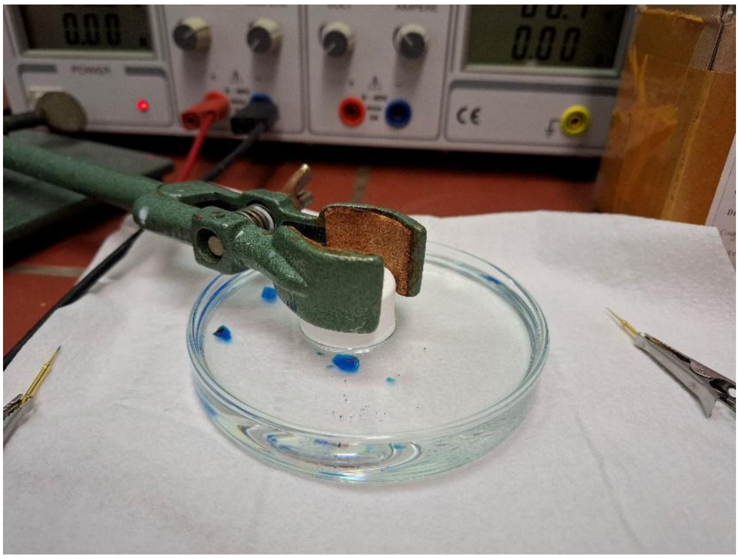

Figure S11. Photograph of a $CuSO_4\ 5{\cdot}H_2O$ crystal in toluene covered by a plastic head space cap equipped with a septum for carrying out a GC-MS based analysis of the gas formed upon electrolysis.

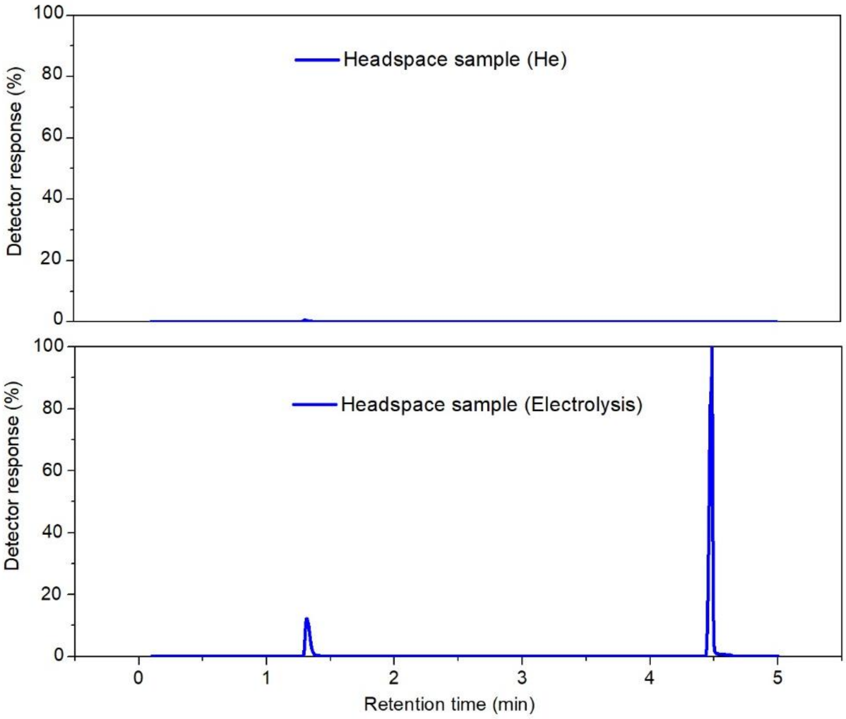

Figure S12. The result of the gas chromatography experiment. Detector response versus retention time plots for a headspace sample **(a)** taken after purging with helium and **(b)** taken after electrolysis for 200 s.

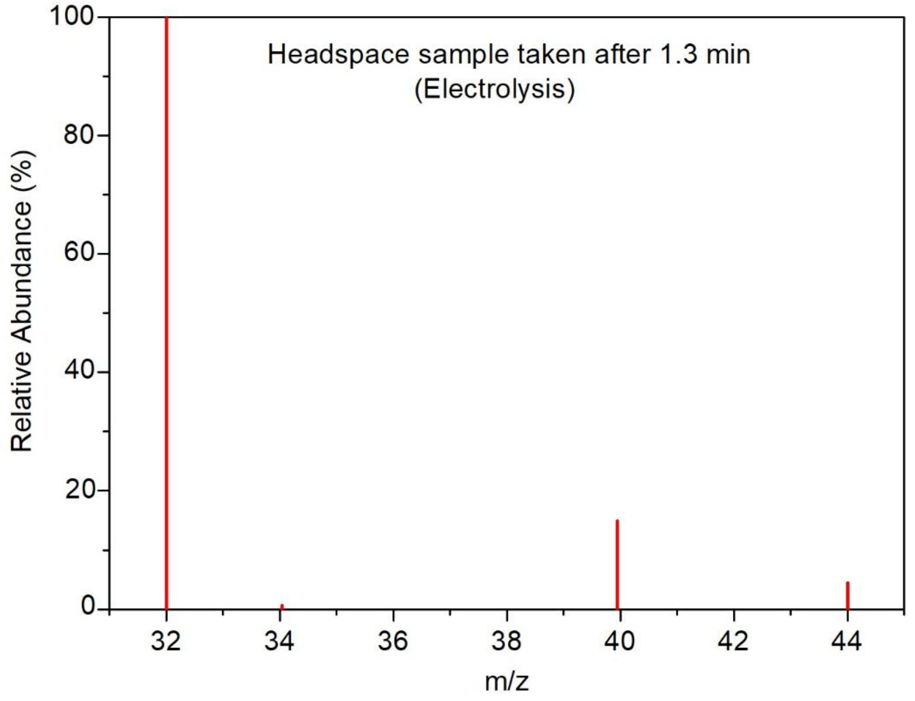

Figure S13. Mass spectrum of the headspace sample (retention time: 1.3 min) taken after electrolysis for 200s.

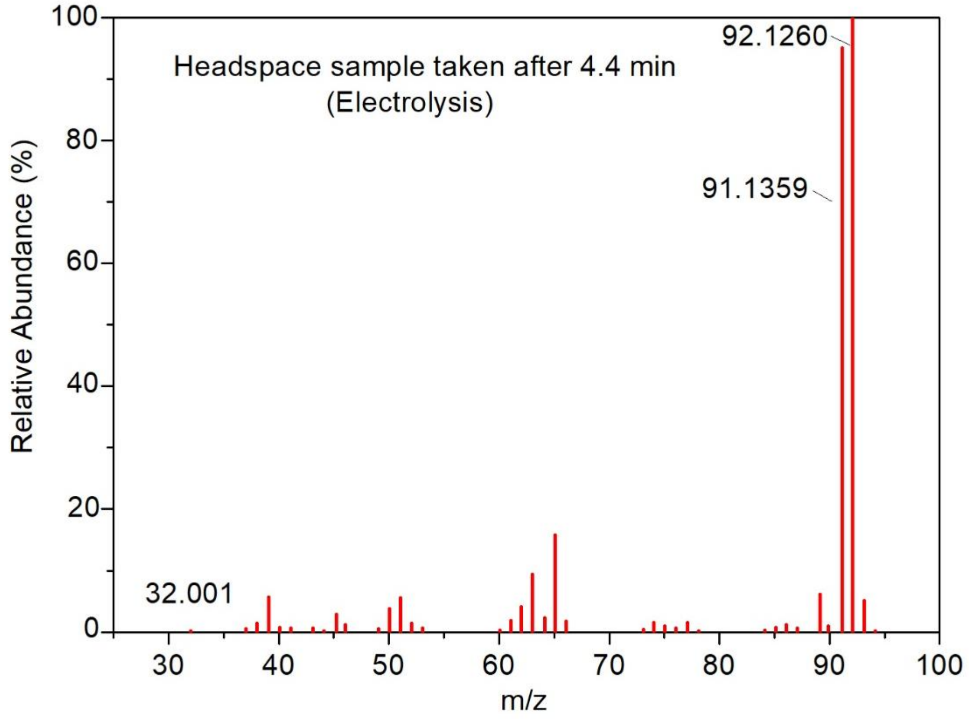

Figure S14. Mass spectrum of the headspace sample (retention time: 4.4 min) taken after electrolysis for 200s.

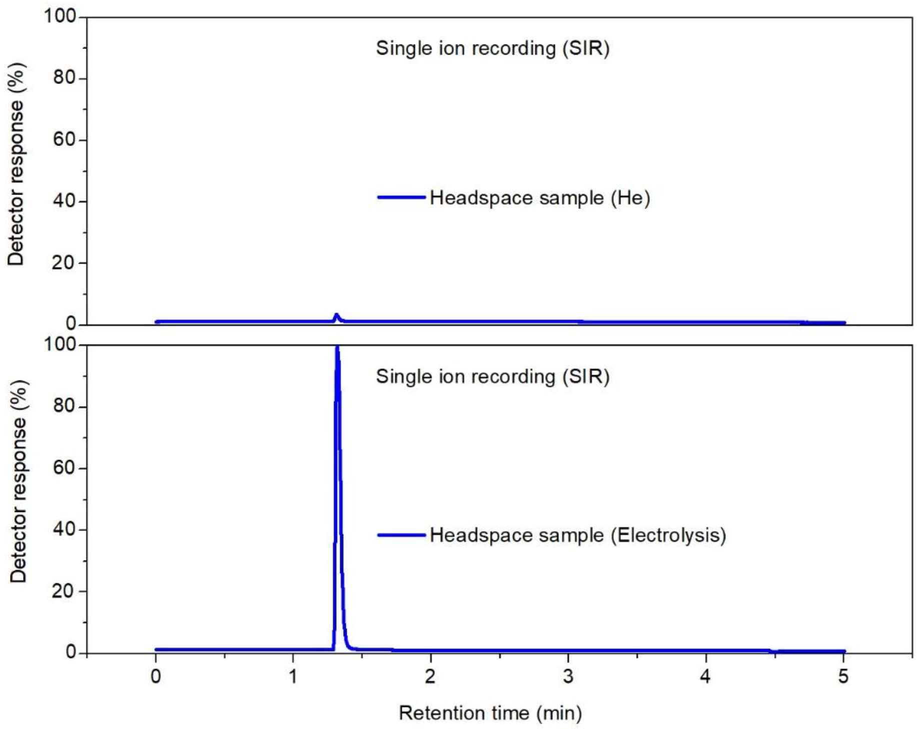

Figure S15. Single ion recording (SIR) signal versus retention time for **(a)** a headspace sample after helium purging and **(b)** a headspace sample after electrolysis for 200 s.

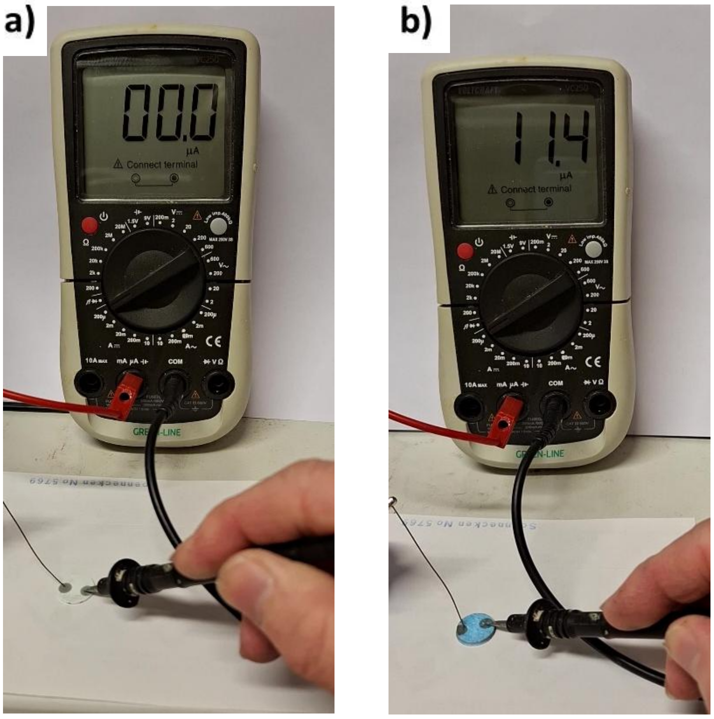

Figure S16. Electric conductivity tests carried out with tablets made from **(a)** anhydrous $CuSO_4$ and **(b)** $CuSO_4 \cdot 5\ H_2O$. A voltage of 8 V was applied to the two gold contacts. Power source:Voltcraft VLP-2403.

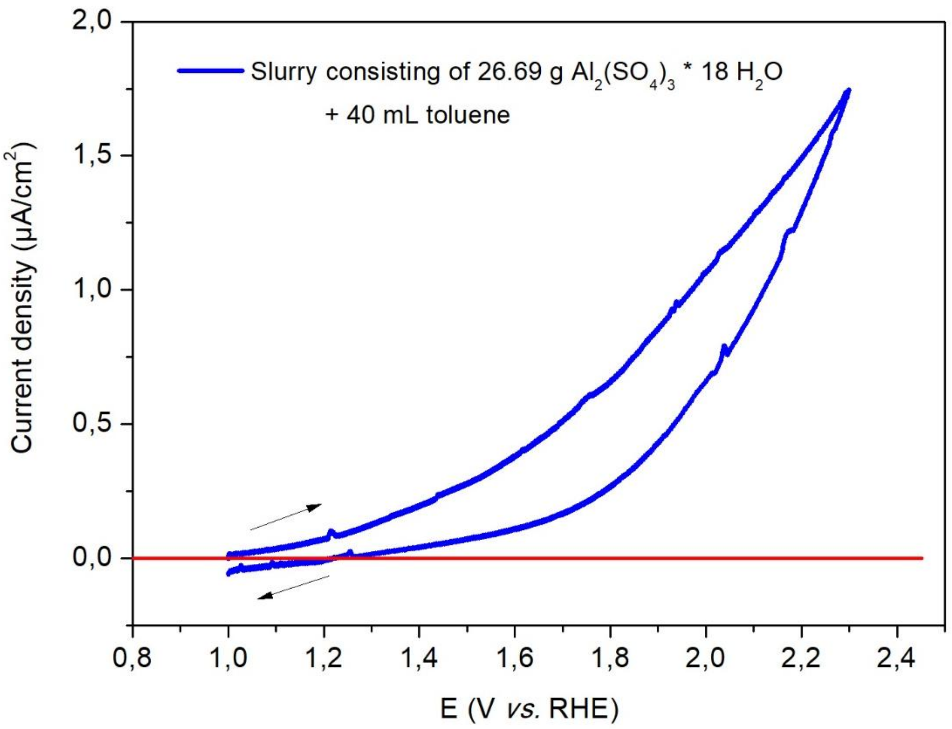

Figure S17. Cyclic voltammogram of the $Al_2(SO_4)_3 \cdot 18\ H_2O$ /toluene slurry. Scan rate: 20 mV/s. Electrode material: Pt (WE), Pt (CE.

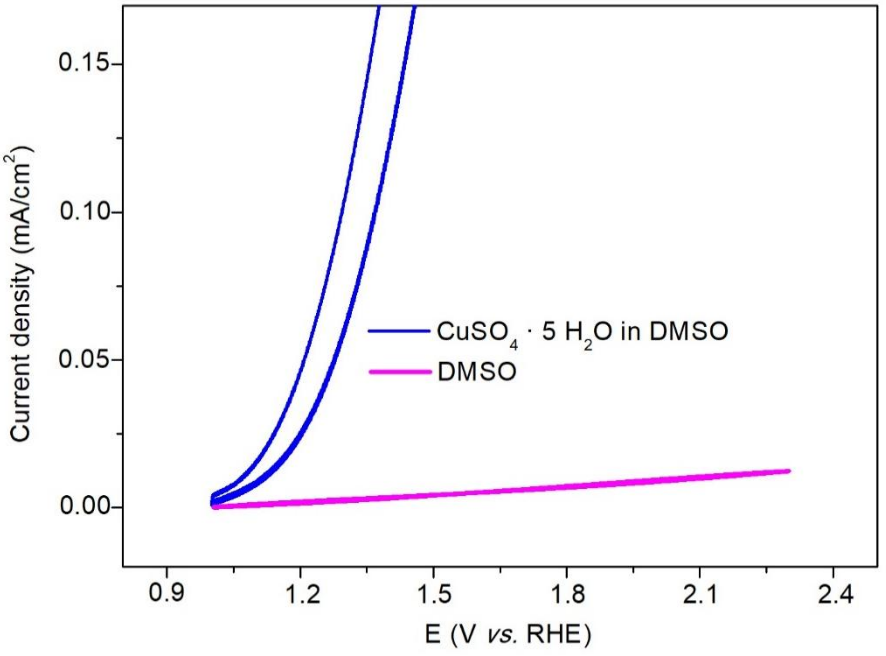

Figure S18. Comparison of the Cyclic voltammograms of DMSO (magenta curve) and the $CuSO_4 \cdot 5\ H_2O$/DMSO slurry (blue curve). WE= Pt (A=4 cm^2); CE= Pt (A=3 cm^2). Potentiostat: Keithley/Tektronix 2460 SourceMeter.

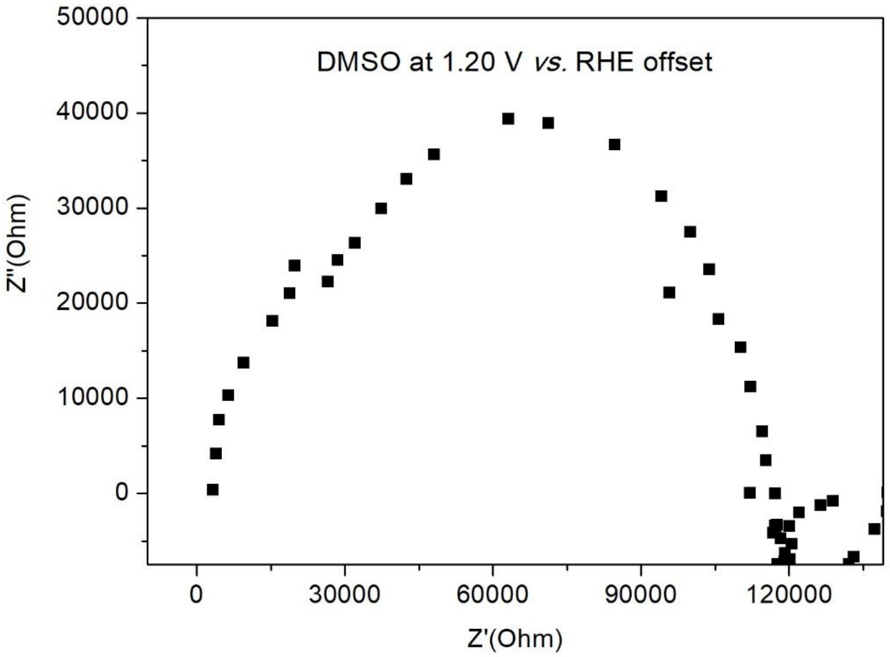

Figure S19. Nyquist plot of the frequency response analysis of DMSO at an offset potential of 1.2 V vs. RHE. WE= Pt (A=3 cm^2); CE= Pt (A=4 cm^2).

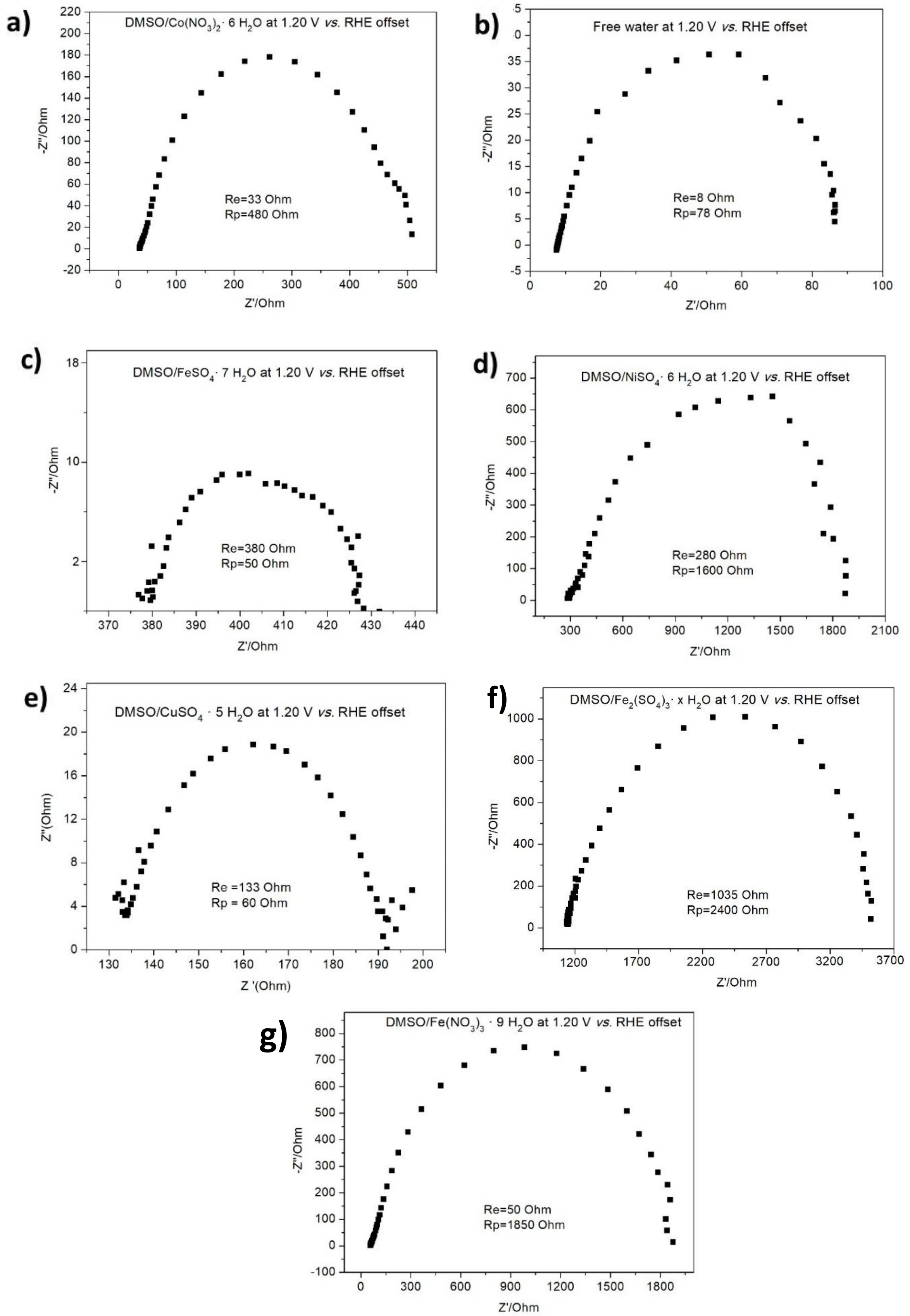

Figure S20. Nyquist plot of the frequency response analysis of DMSO-based suspensions with **(a)** $Co(NO_3)_2·6\ H_2O$, **(c)** $FeSO_4·7\ H_2O$, **(d)** $NiSO_4·6\ H_2O$, **(e)** $CuSO_4·5\ H_2O$, **(f)** $Fe_2(SO_4)_3·x\ H_2O$, **(g)** $Fe(NO_3)_3·9\ H_2O$ slurry togther with **(b)** free water at an offset potential of 1.2 V vs. RHE. WE= Pt (A=3 cm^2); CE= Pt (A=4 cm^2).

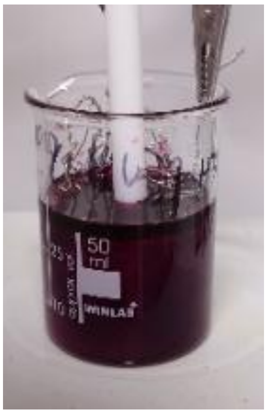

Figure S21. Picture of $Co(NO_3)_2 \cdot 6\ H_2O$ suspended in DMSO.

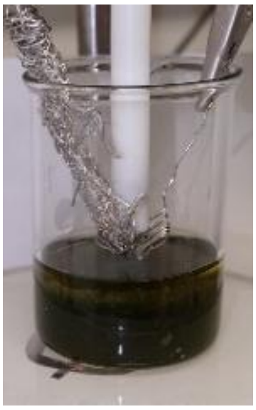

Figure S22. Picture of $FeSO_4 \cdot 7\ H_2O$ suspended in DMSO.

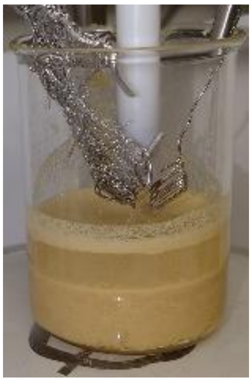

Figure S23. Picture of $Fe_2(SO_4)_3 \cdot x\ H_2O$ suspended in DMSO.

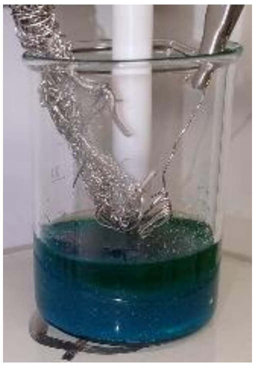

Figure S24. Picture of $CuSO_4 \cdot 5\ H_2O$ suspended in DMSO.

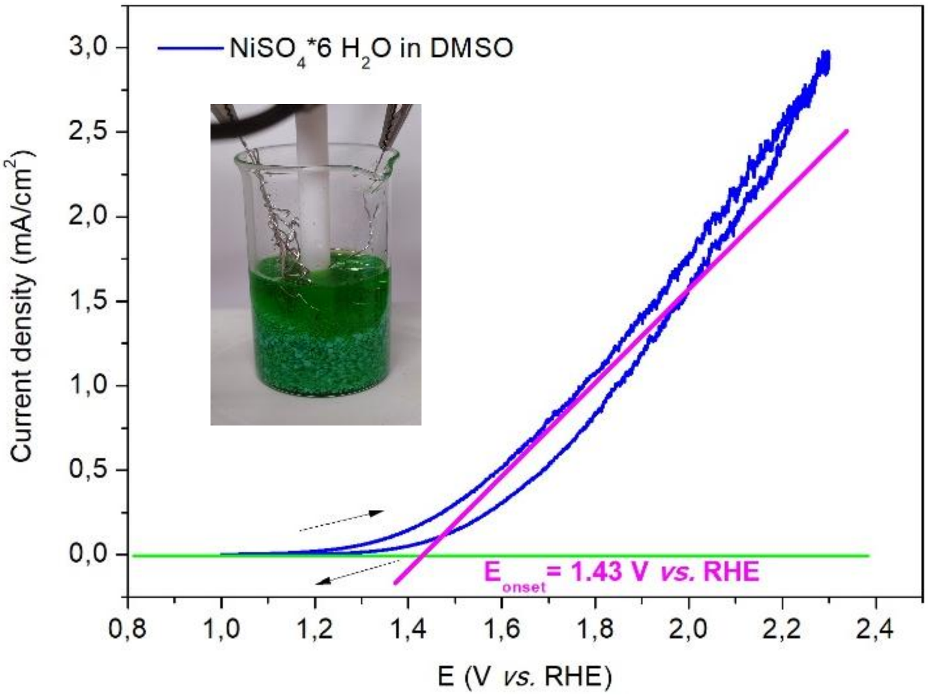

Figure S25. CV measurement of a suspension of hydrated $NiSO_4$ in DMSO. The inset picture shows the corresponding slurry. WE= Pt (A=4 cm^2); CE= Pt (A=3 cm^2).

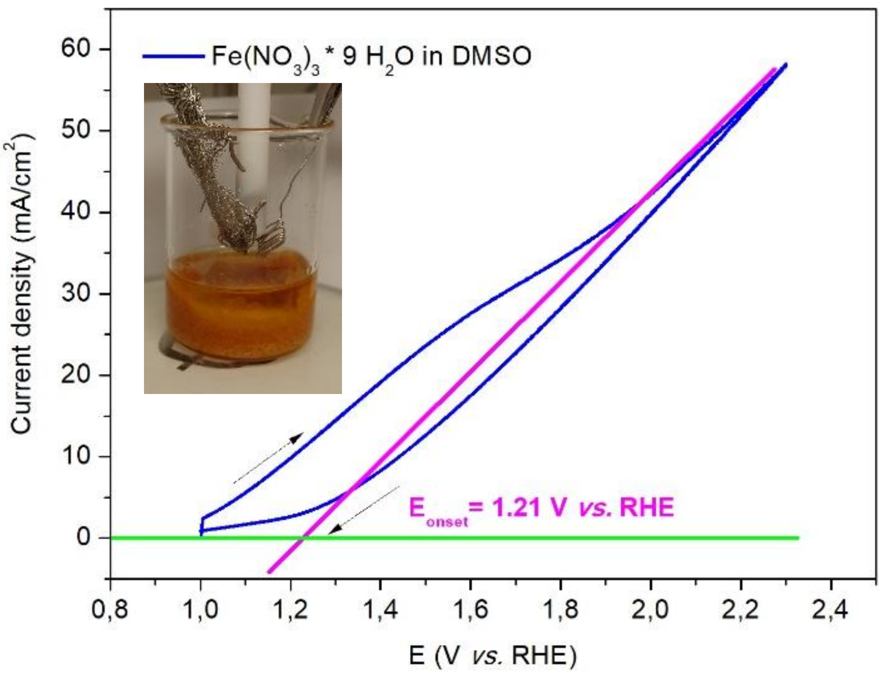

Figure S26. CV measurement of a suspension of hydrated $Fe(NO_3)_3$ in DMSO. The inset picture shows the corresponding slurry. WE= Pt (A=4 cm^2); CE= Pt (A=3 cm^2).

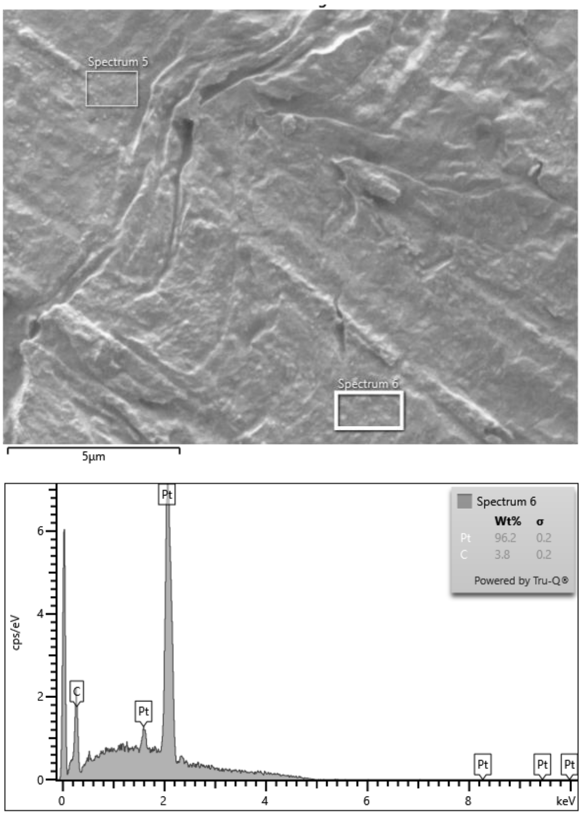

Figure S27. The results from EDS experiments carried out with unused Pt. Detector: secondary electron detector; Acceleration voltage: 5 kV; working distance: 5 mm.

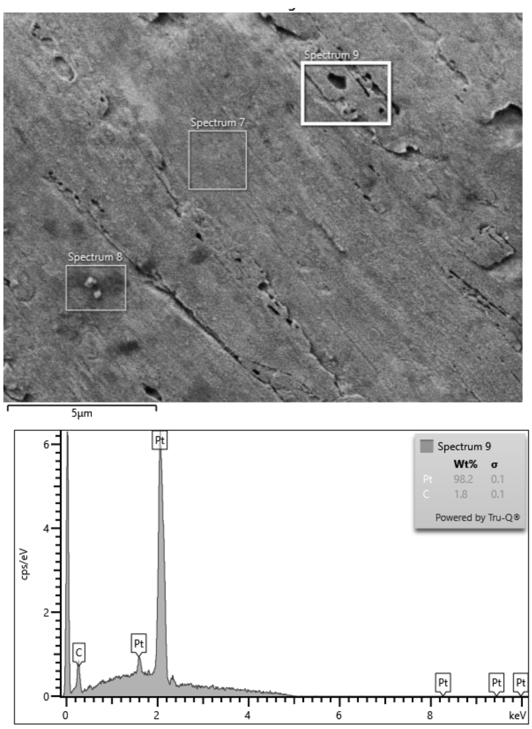

Figure S28. The results from EDS experiments carried out with Pt used as a working electrode for the electrolysis of 17.38 g (59. 7 mmol) of $Co(NO_3)_2 \cdot 6\ H_2O$ in 20 mL of DMSO. Detector: secondary electron detector; Acceleration voltage: 5 kV; working distance: 5 mm.

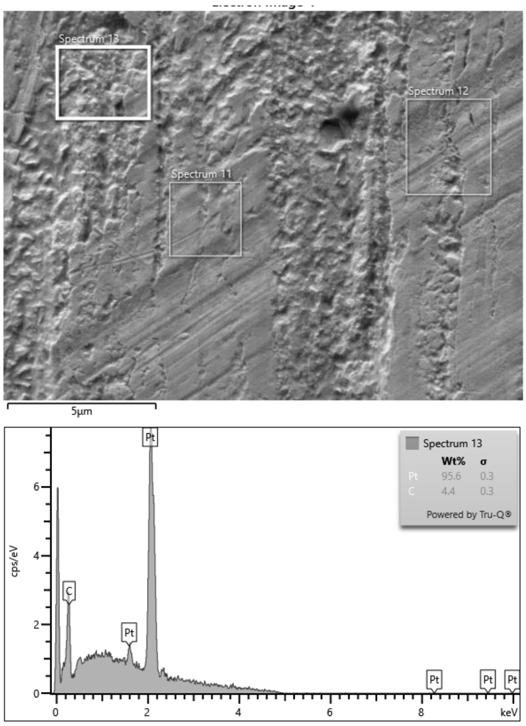

Figure S29. The results from EDS experiments carried out with Pt used as a working electrode for the electrolysis of 20 g $CuSO_4 \cdot 5\ H_2O$ (80.1 mmol) in 22 mL DMSO. Detector: secondary electron detector; Acceleration voltage: 5 kV; working distance: 5 mm.

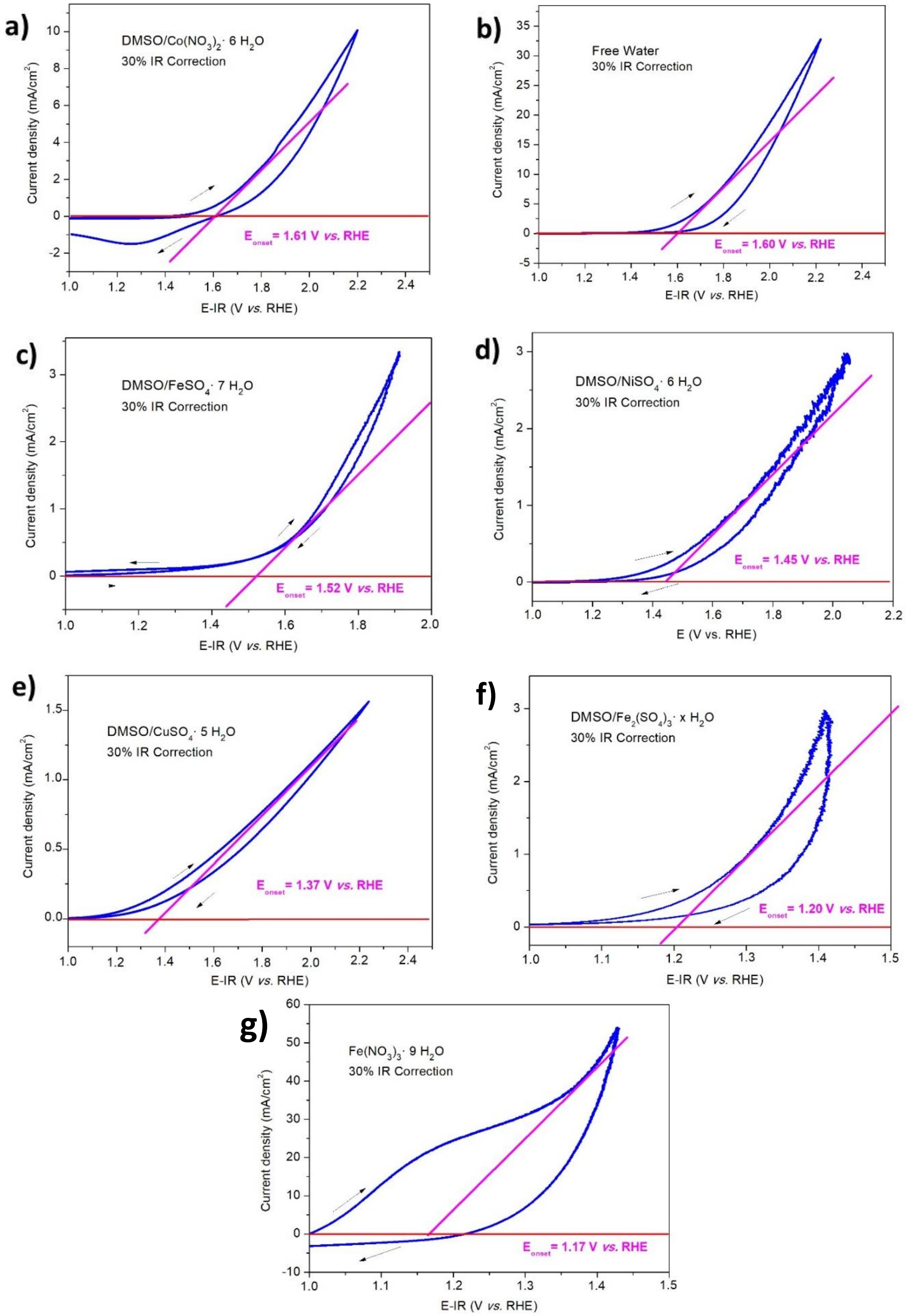

Figure S30. Voltage drop compensation (based on electrolyte resistance derived from EIS experiments (Figure S20) applied to cyclic voltammetry measurements performed with **(a-g)** hydrated salt/DMSO suspensions and **(b)** pH 7 corrected 0.1 molar (K_2HPO_4/KH_2PO_4) buffer solution. Platinum was chosen as WE and CE; electrode area: 1 cm^2 (WE), 3 ×4 cm geometric area (CE).

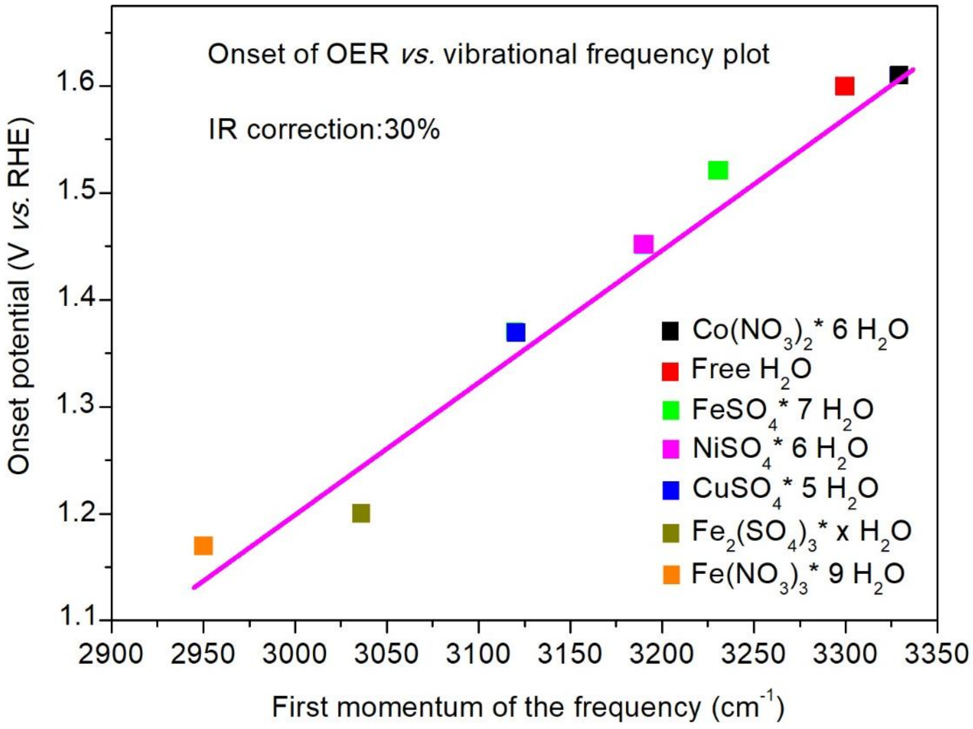

Figure S31. The correlation between the onset potential for the oxygen evolution reaction as defined by the tangent method. The onset potential is derived from IR corrected CV data (30% IR correction based on electrolyte resistance Figure S29, S19).

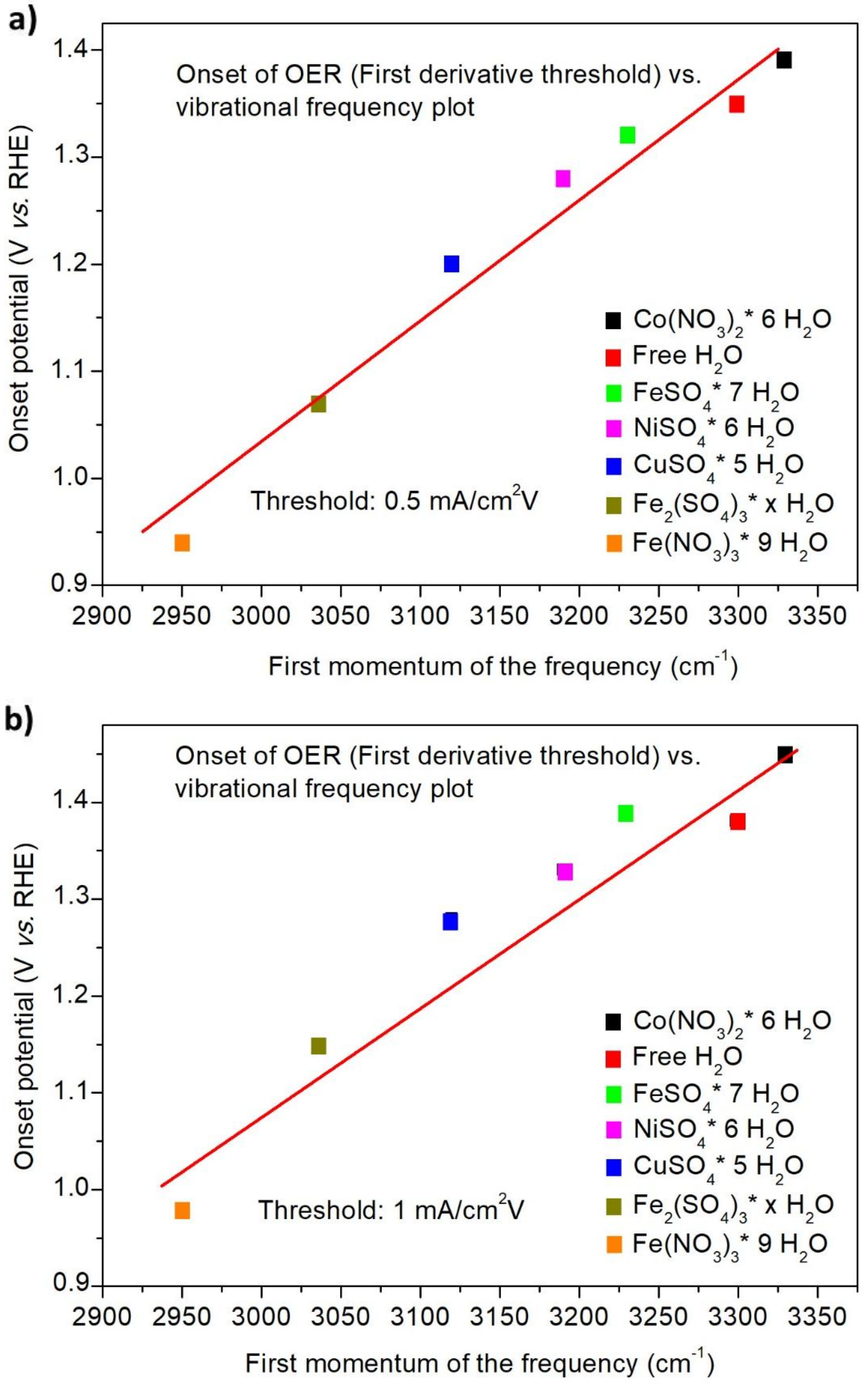

Figure S32. The correlation between the onset potential for the oxygen evolution reaction as defined by the first derivative method, *i.e.* a threshold of (**a)** 0.5 mA/cm^2 V, (**b)** 1.0 mA/cm^2 V for the first derivative of the CV curve, was used to define the onset potential.

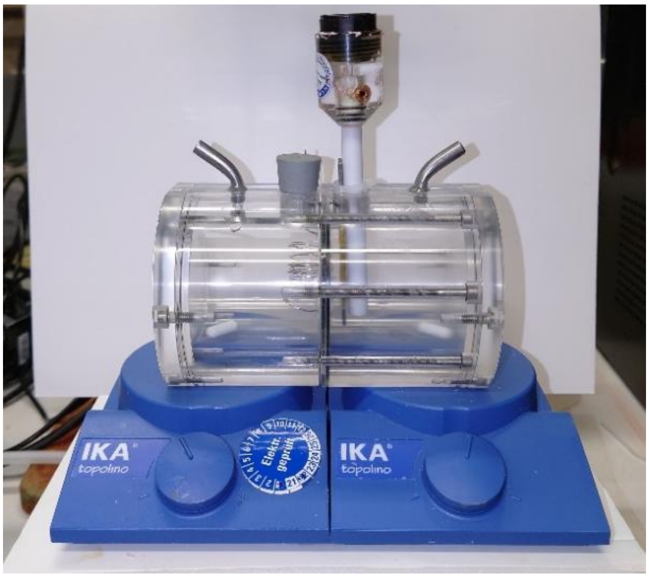

Figure S33. Photo of the homemade electrolysis cell. Dimensions: 12.5 x 8.2 x 7.5 cm (length x width x height); inner diameter: 5 cm; volume: 220 cm^3. Material: acrylic glass.

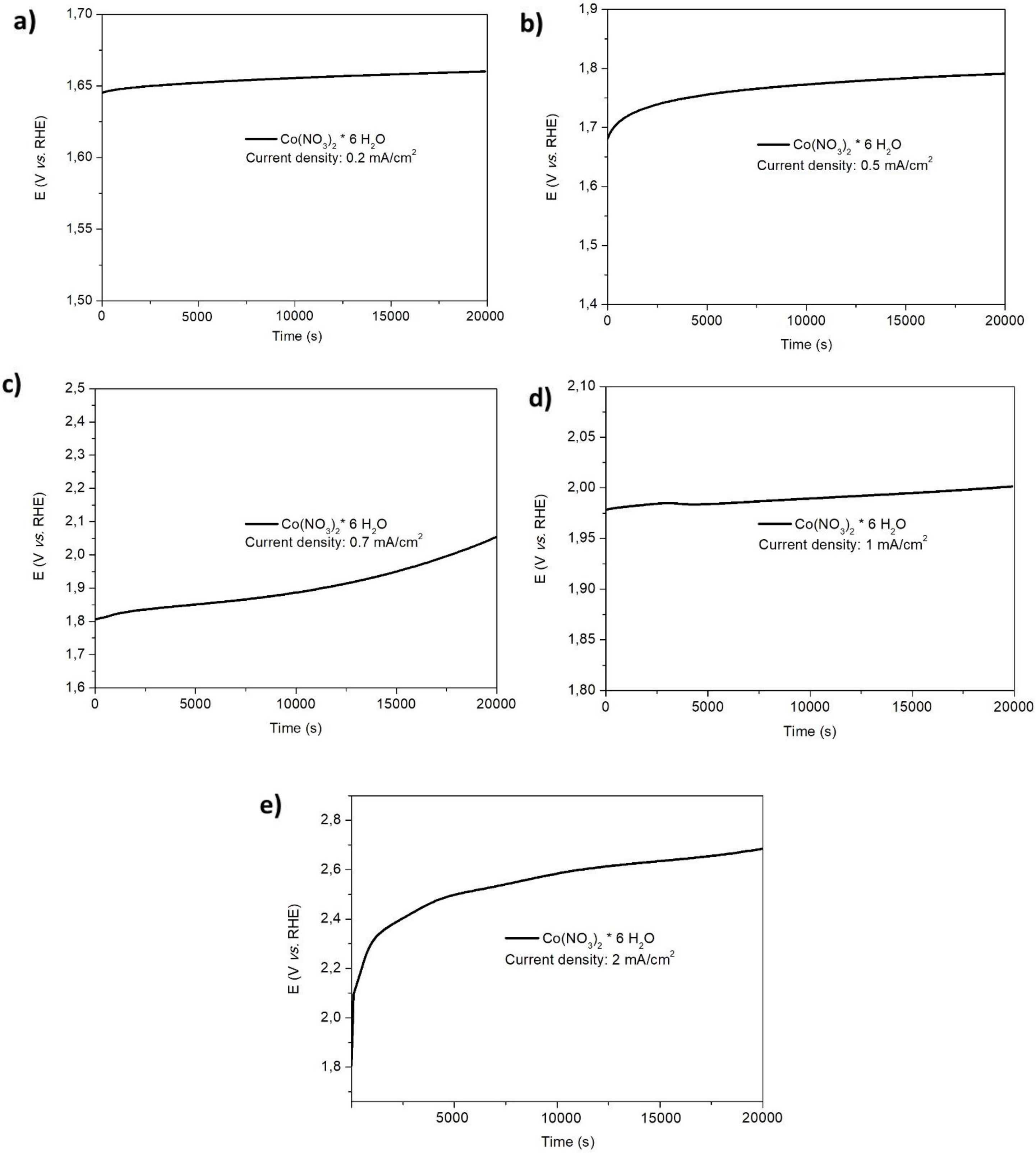

Figure S34. The outcome from galvanostatic tests carried out with $Co(NO_3)_2{\cdot}6\ H_2O$/DMSO suspensions. WE= Pt (A=1 cm^2); CE= Pt (A=12 cm^2).

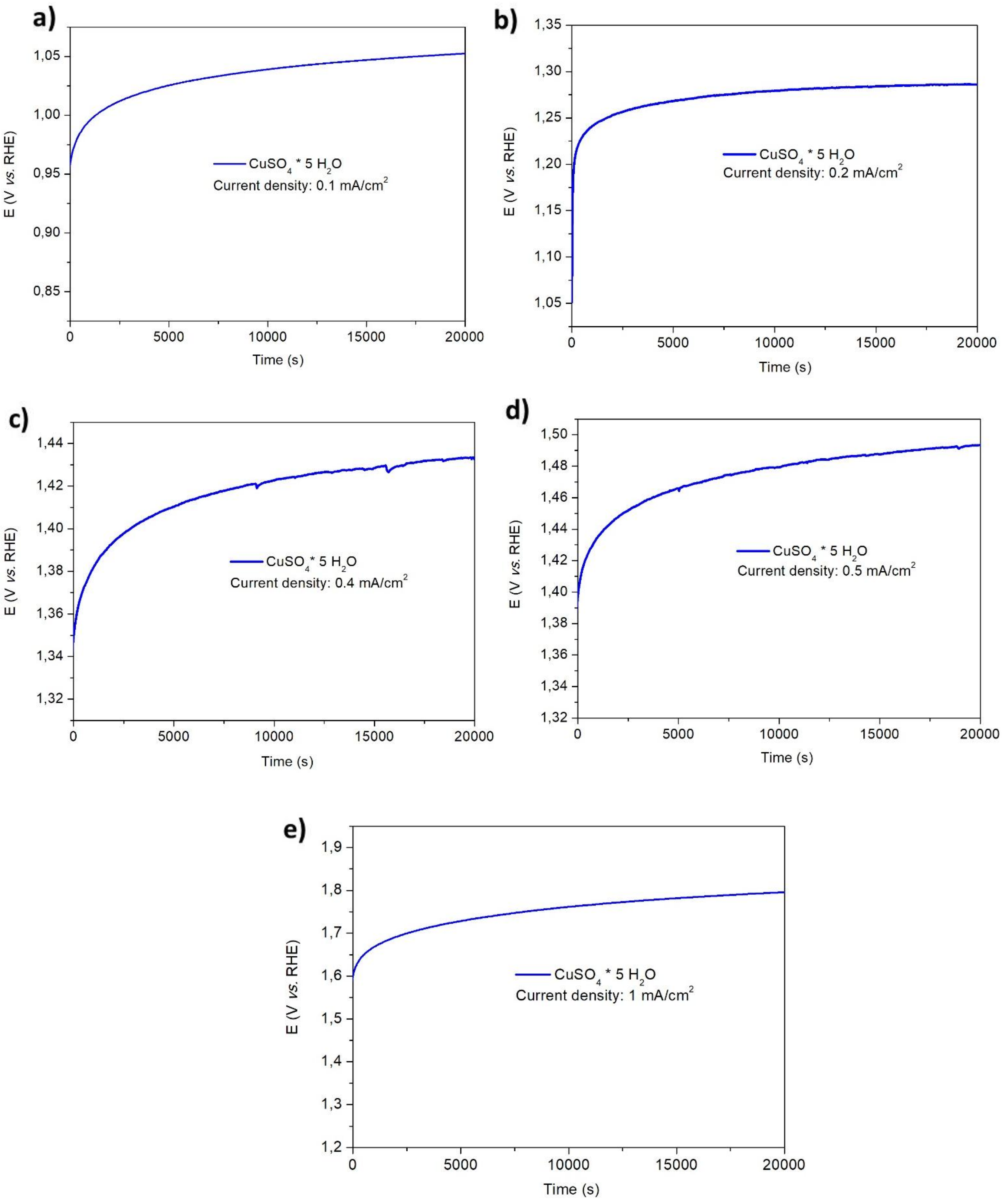

Figure S35. The results of the galvanostatic tests carried out with $Cu(SO_4)_2{\cdot}5\ H_2O$/DMSO suspensions. Electrode area (WE): 1 cm^2. WE= Pt (A=1 cm^2); CE= Pt (A=12 cm^2).

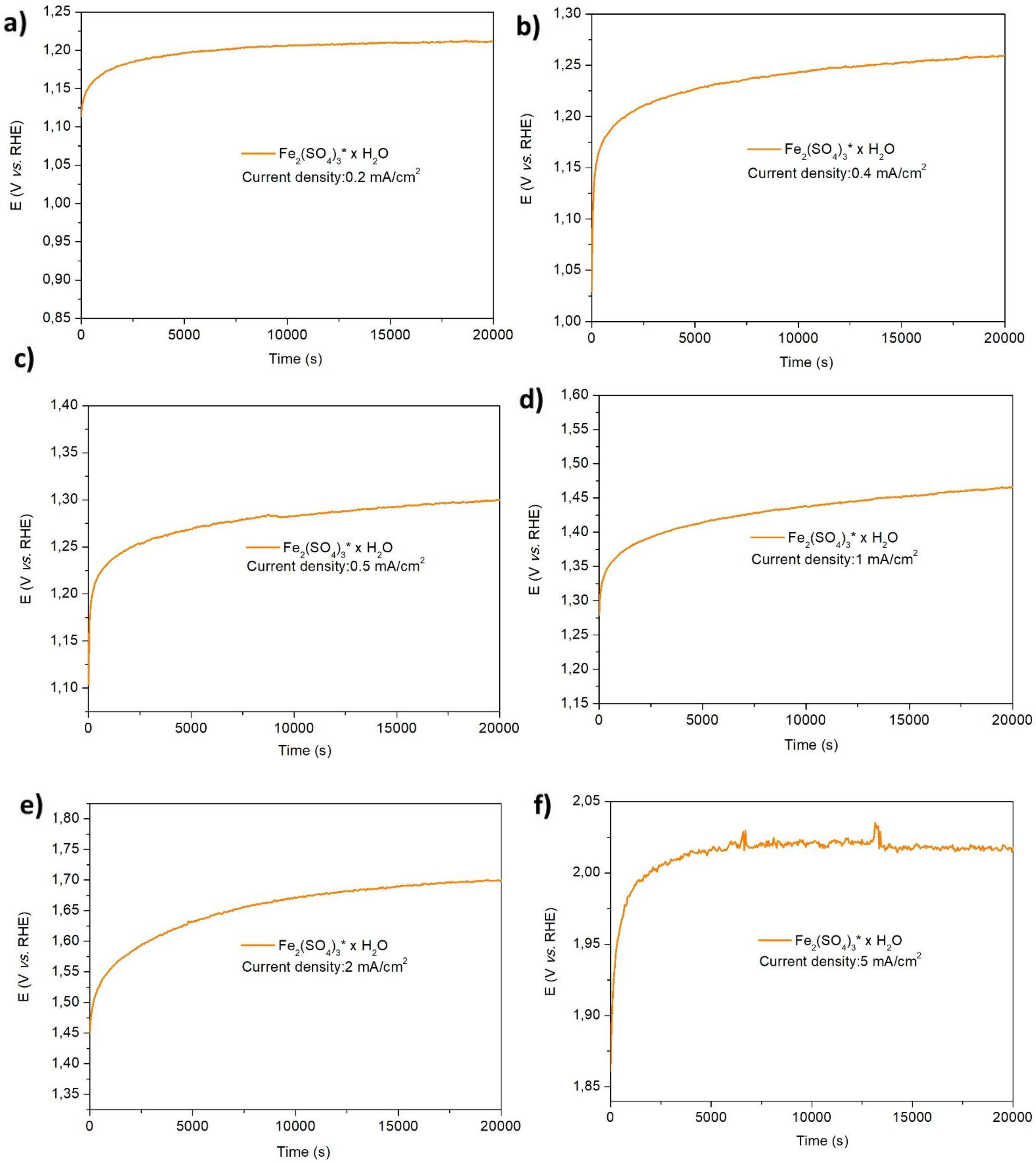

Figure S36. The results of the galvanostatic tests carried out with $Fe_2(SO_4)_3 \cdot X\ H_2O$/DMSO suspensions. Electrode area (WE): 1 cm^2. WE= Pt (A=1 cm^2); CE= Pt (A=12 cm^2).

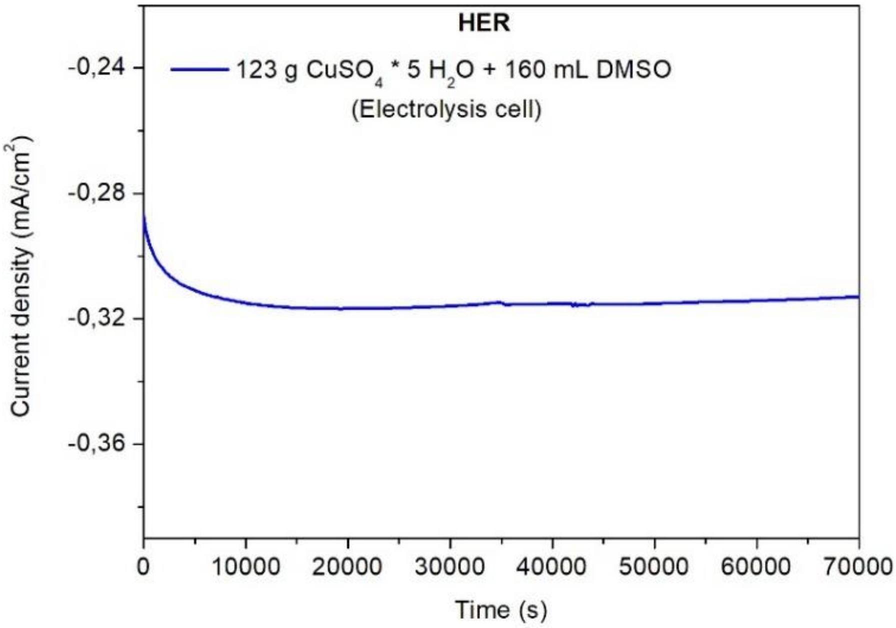

Figure S37. CP plot derived from a $CuSO_4 \cdot 5\ H_2O$/DMSO suspension at a current density of 1 mA/cm^2. WE= Pt (A=1 cm^2); CE= Pt (A=12 cm^2).

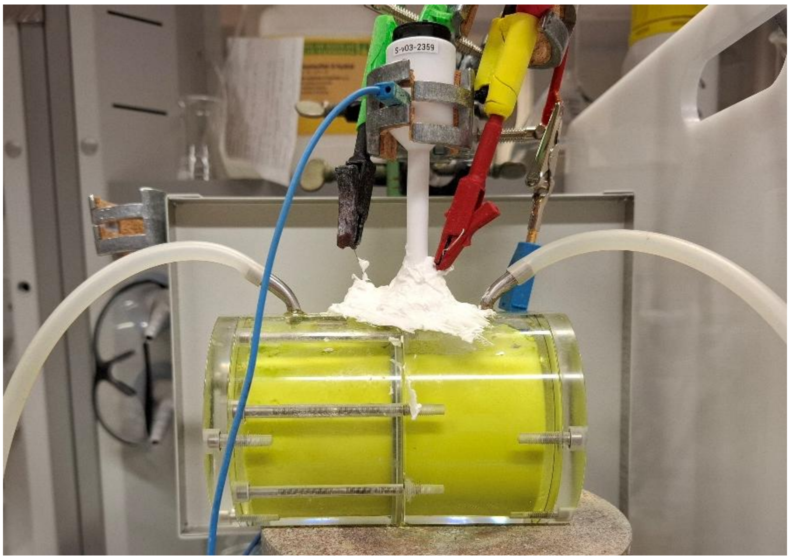

Figure S38. Setup used to determine the faradaic efficiency. The electrolysis cell was filled with a suspension consisting of 189.9 g $Fe(NO_3)_3 \cdot 9\ H_2O$ and 171 ml DMSO. Electrodes: Pt (CE and WE). Current density 300 mA/cm^2; T=296.15 K. Compartments separated by a Nafion® 212 membrane.

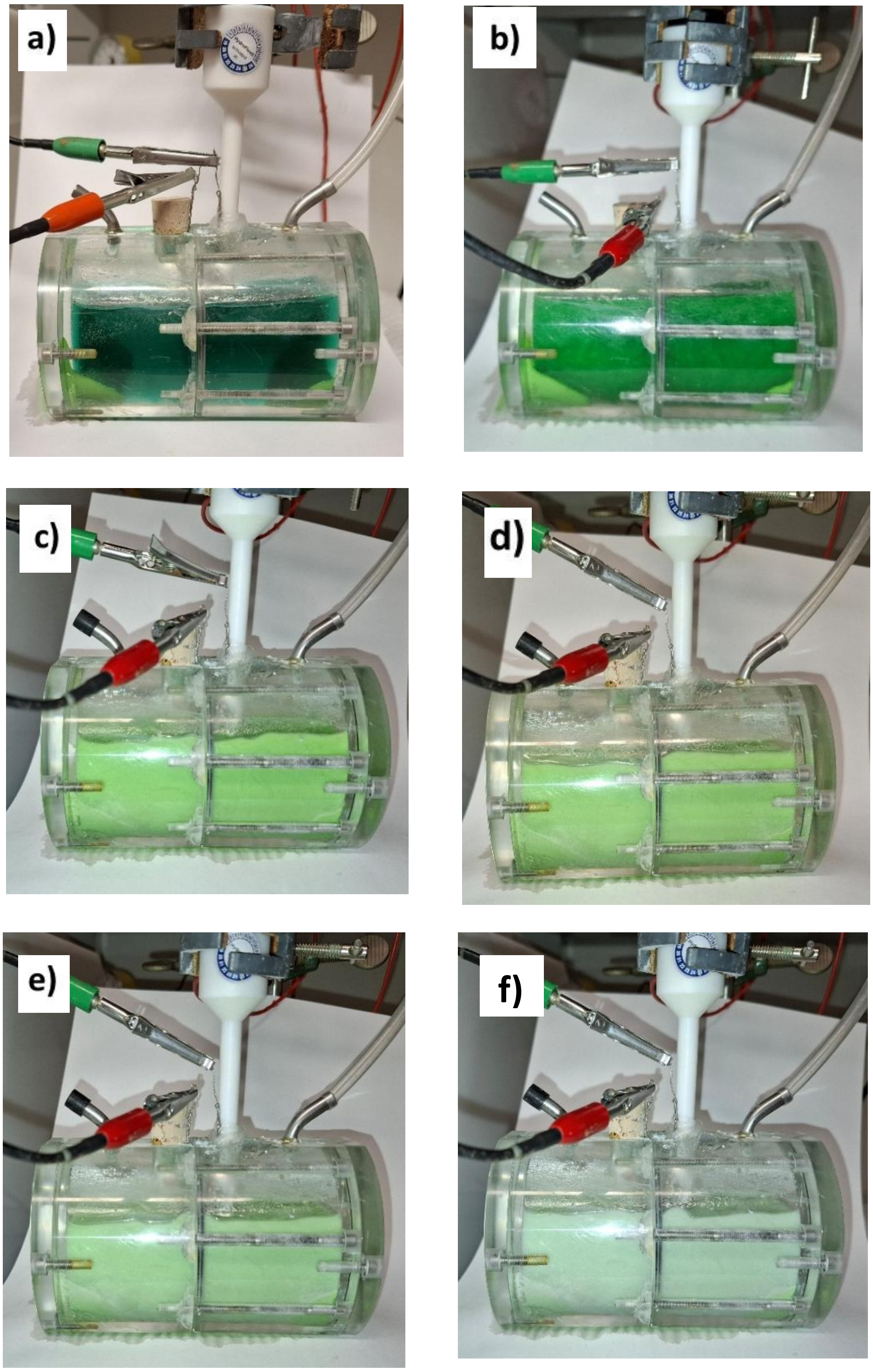

Figure S39. Electrolysis of an electrolyte comprising 215 mL of DMSO and 6 g of $CuSO_4 \cdot 5\ H_2O$ at a total current of 60 mA (j=20 mA/cm^2); Pt WE (A=3 cm^2) + Pt CE (A=3 cm^2) at operating times of 0h (a), 7 h (b), 16 h (c), 25 h (d), 51 h (e), 145 h (f).

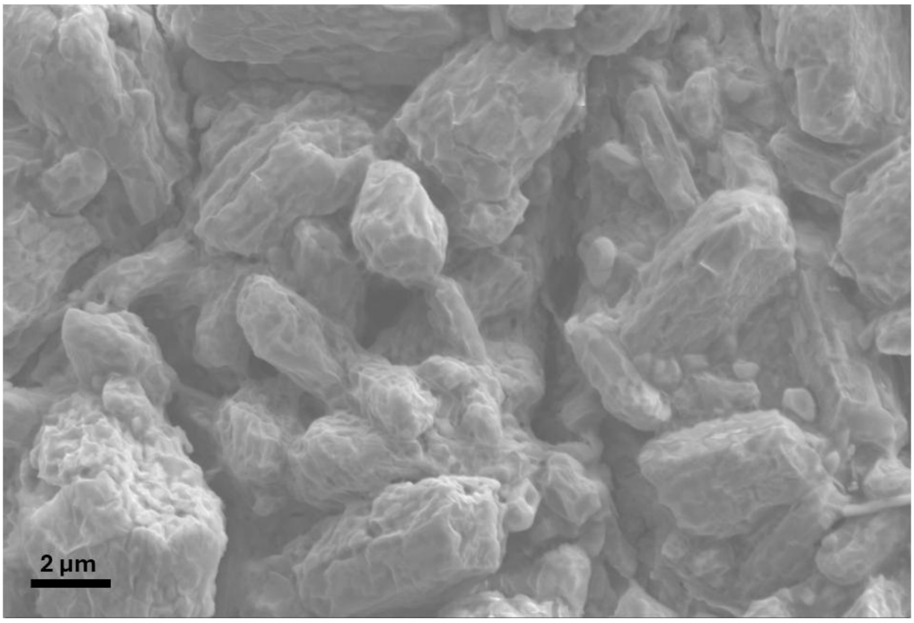

Figure S40. SEM image of $CuSO_4 \cdot 5\ H_2O$ stirred for 167 h in DMSO. The acceleration voltage was set to 5 kV. The SEM images were taken with a secondary electron detector.

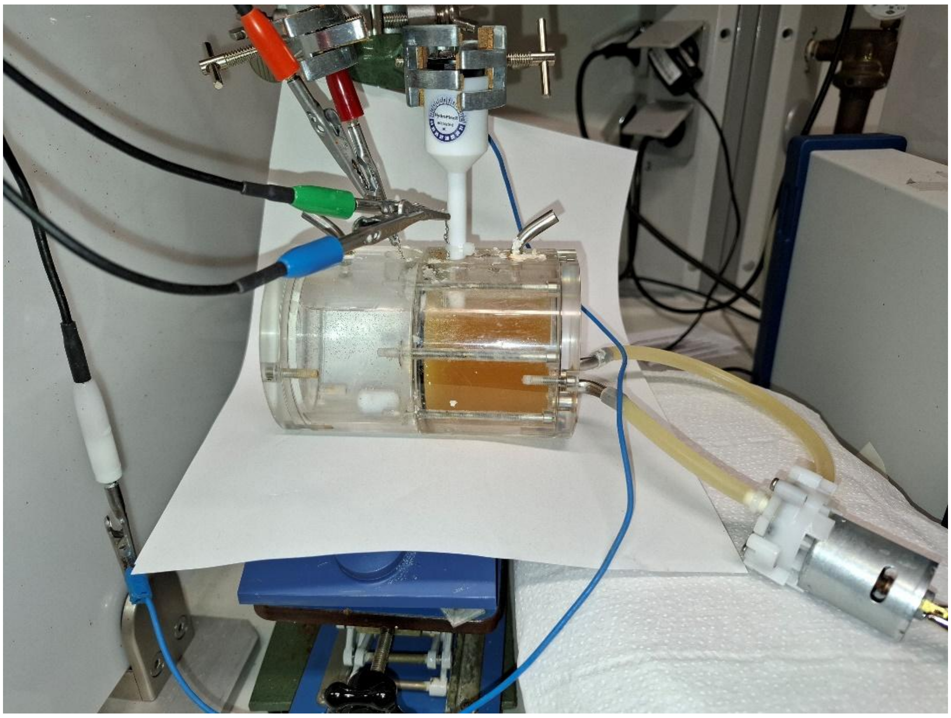

Figure S41.Image of the electrolysis cell used for the electrolysis of aqueous solutions equipped with a pump for circulating the anolyte . Pt WE: 3 cm^2; Pt CE: 4 cm^2.

Anolyte	Cell voltage in V at j (mA cm^{-2})	OER in mV at j (mA cm^{-2})
i (0.125 M H_2SO_4)	2.29 (100)	81 (100)
i (0.125 M H_2SO_4)	2.43 (150)	151 (150)
i (0.125 M H_2SO_4)	2.52 (200)	211 (200)
i (0.125 M H_2SO_4)	2.57 (225)	241 (225)
i (0.125 M H_2SO_4)	2.68 (300)	291 (300)
i (0.125 M H_2SO_4)	2.80 (375)	341 (375)
ii (H_2SO_4/$Fe_2(SO_4)_3 \cdot H_2O$)	2.14 (100)	22 (100)
ii (H_2SO_4/$Fe_2(SO_4)_3 \cdot H_2O$)	2.23 (150)	45 (150)
ii (H_2SO_4/$Fe_2(SO_4)_3 \cdot H_2O$)	2.31 (200)	67 (200)
ii (H_2SO_4/$Fe_2(SO_4)_3 \cdot H_2O$)	2.35 (225)	86 (225)
ii (H_2SO_4/$Fe_2(SO_4)_3 \cdot H_2O$)	2.42 (300)	100 (300)
ii (H_2SO_4/$Fe_2(SO_4)_3 \cdot H_2O$)	2.51 (375)	151 (375)
iii (H_2SO_4/Na_2SO_4)	2.30 (100)	51 (100)
iii (H_2SO_4/Na_2SO_4)	2.42 (150)	131 (150)
iii (H_2SO_4/Na_2SO_4)	2.53 (200)	181 (200)
iii (H_2SO_4/Na_2SO_4)	2.59 (225)	231 (225)
iii (H_2SO_4/Na_2SO_4)	2.74 (300)	311 (300)
iii (H_2SO_4/Na_2SO_4)	2.90 (375)	381 (375)

Table S2. Results from water electrolysis tests carried out in an electrolysis cell (Figure S41) upon using 3 M H_2SO_4 as the catholyte and different anolytes (column I); cell voltage at j=100, 150, 200, 225, 300 and 350 mA/cm^2 (column II), OER overpotential at j=100, 150, 200, 225, 300 and 350 mA cm^{-1}(column III), respectively.

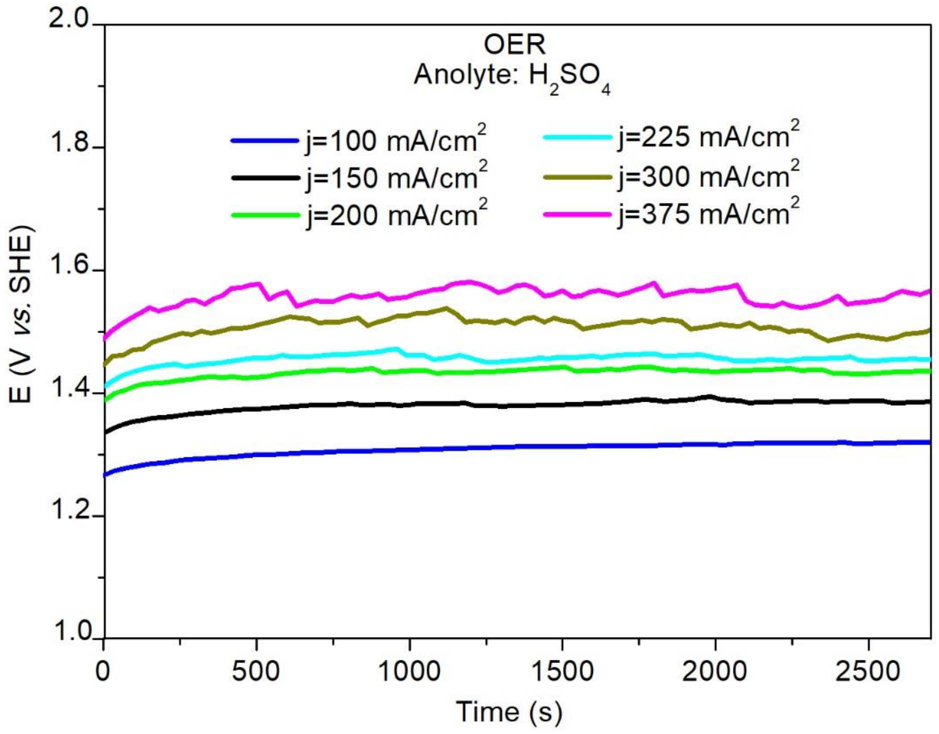

Figure S42. Chronopotentiometry measurements performed with anolyte i at different current densities in a cell (Figure S41) using a Fumasep FAP 450 anion exchange membrane and Pt electrodes as anode and cathode. Pt WE: 3 cm^2; Pt CE: 4 cm^2

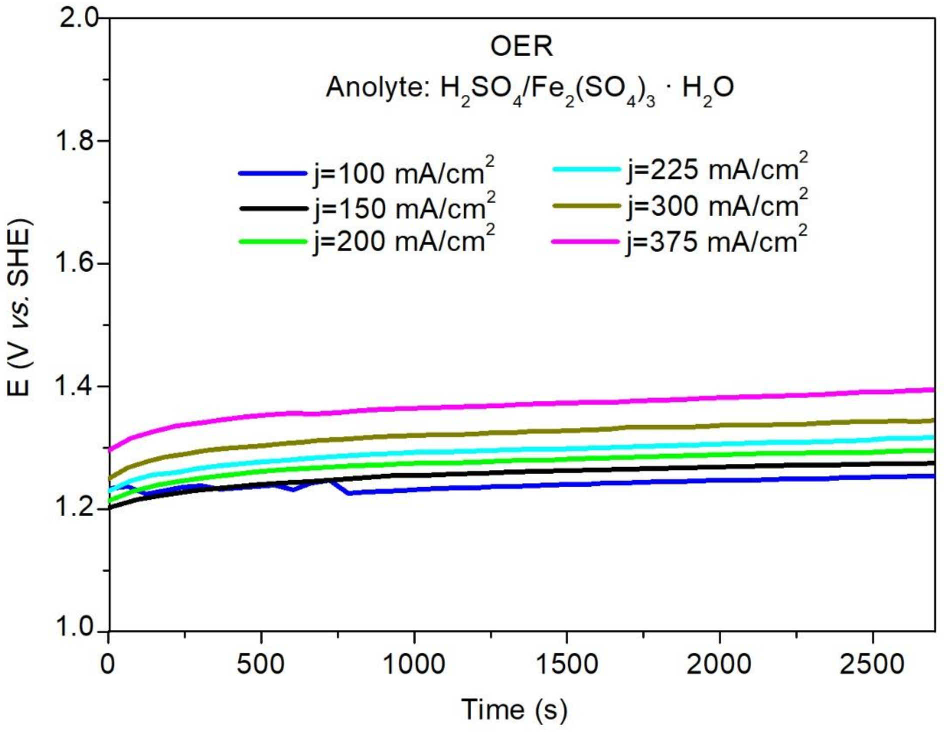

Figure S43. Chronopotentiometry measurements performed with anolyte ii at different current densities in a cell (Figure S41) using a Fumasep FAP 450 anion exchange membrane and Pt electrodes as anode and cathode. Pt WE: 3 cm^2; Pt CE: 4 cm^2

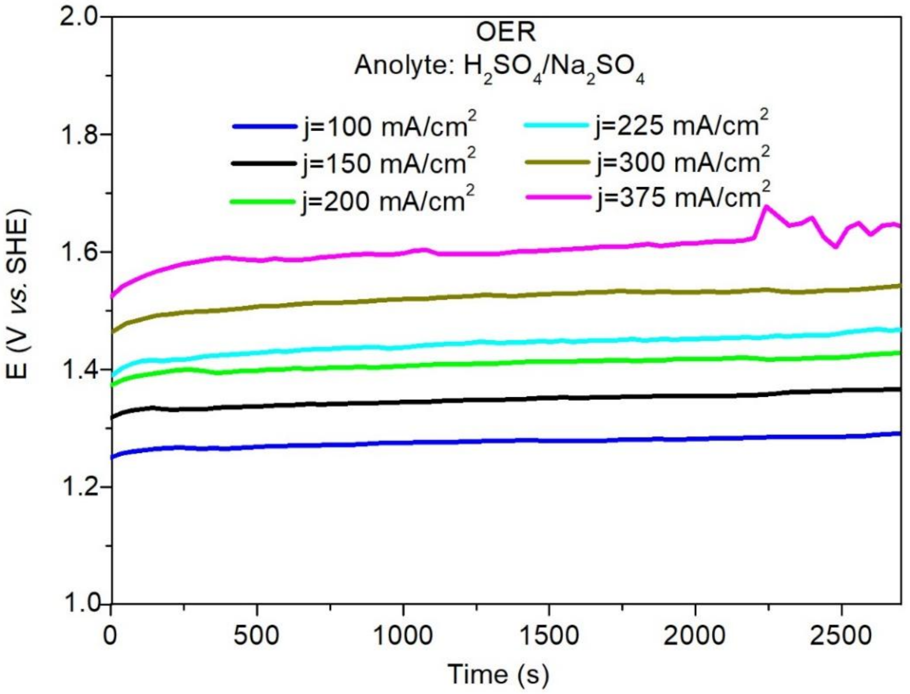

Figure S44. Chronopotentiometry measurements performed with anolyte iii at different current densities in a cell (Figure S41) using a anion exchange membrane Fumasep FAP 450 and Pt electrodes as anode and cathode. Pt WE: 3 cm^2; Pt CE: 4 cm^2

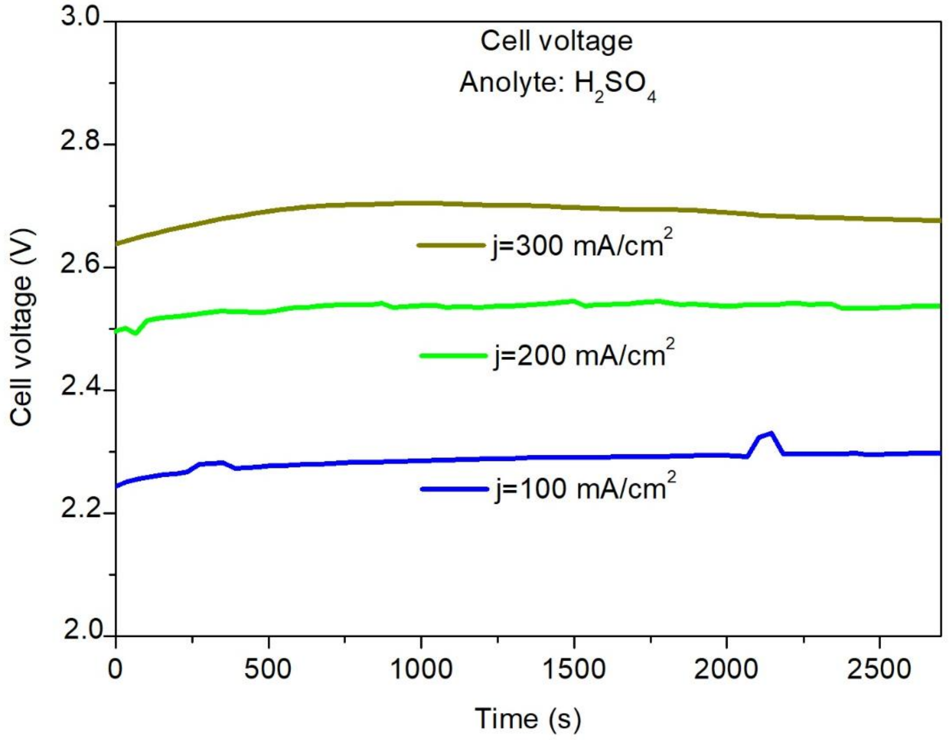

Figure S45. Cell voltage derived from water electrolysis experiments performed with anolyte i at different current densities in a cell (Figure S41) using a anion exchange membrane Fumasep FAP 450 and Pt electrodes as anode and cathode. Pt WE: 3 cm^2; Pt CE: 4 cm^2

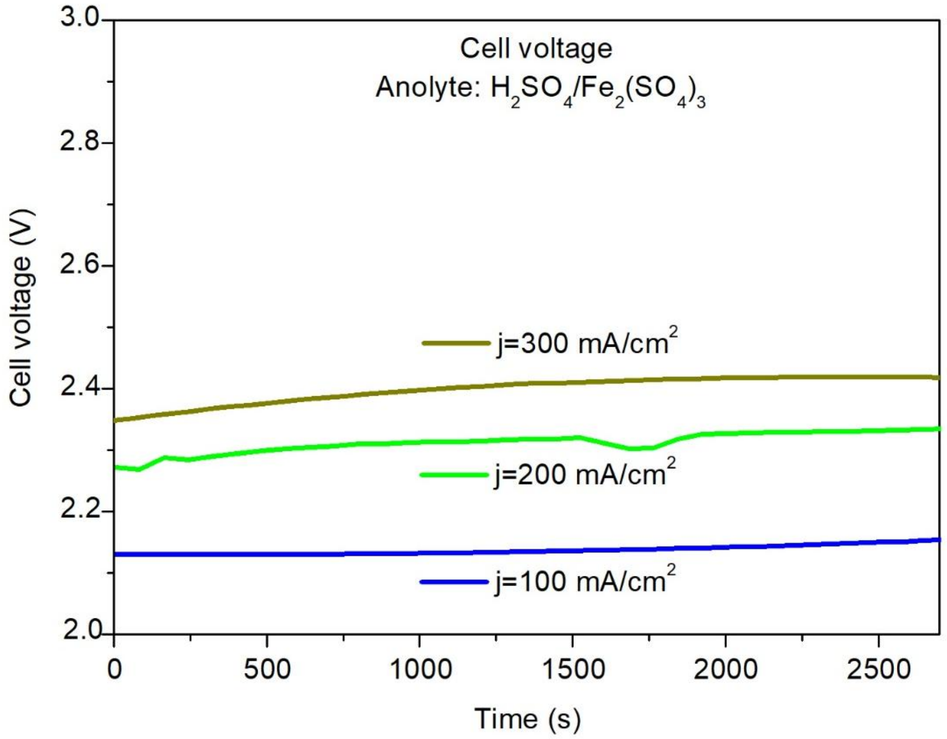

Figure S46. Cell voltage derived from water electrolysis experiments performed with anolyte ii at different current densities in a cell (Figure S41) using a anion exchange membrane Fumasep FAP 450 and Pt electrodes as anode and cathode. Pt WE: 3 cm^2; Pt CE: 4 cm^2

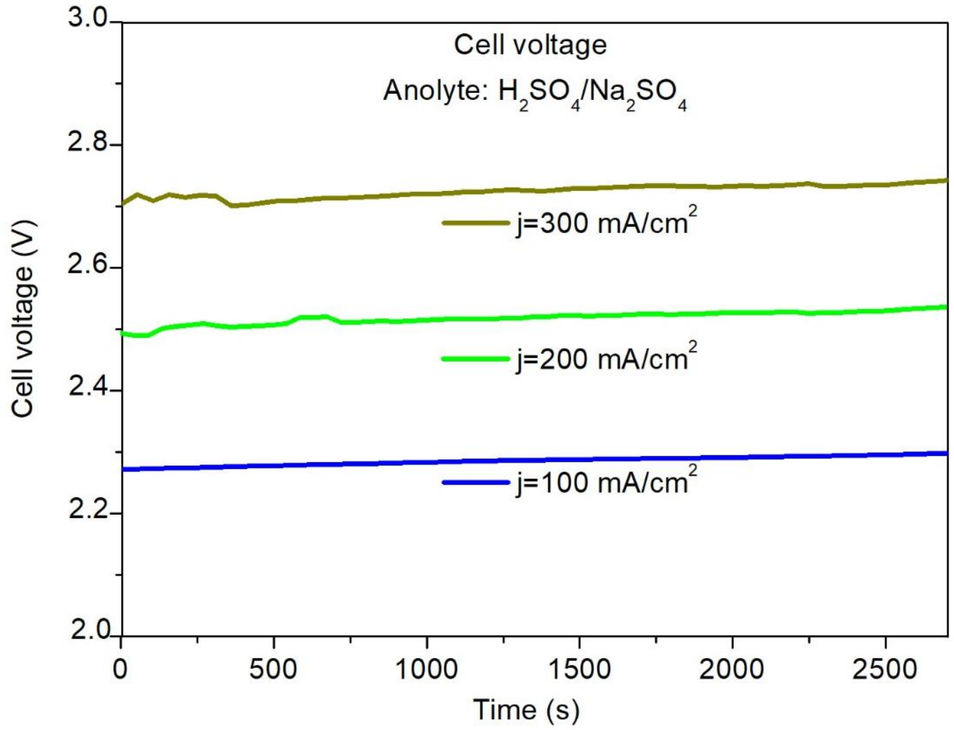

Figure S47. Cell voltage derived from water electrolysis experiments performed with anolyte iii at different current densities in a cell (Figure S41) using a anion exchange membrane Fumasep FAP 450 and Pt electrodes as anode and cathode. Pt WE: 3 cm^2; Pt CE: 4 cm^2.

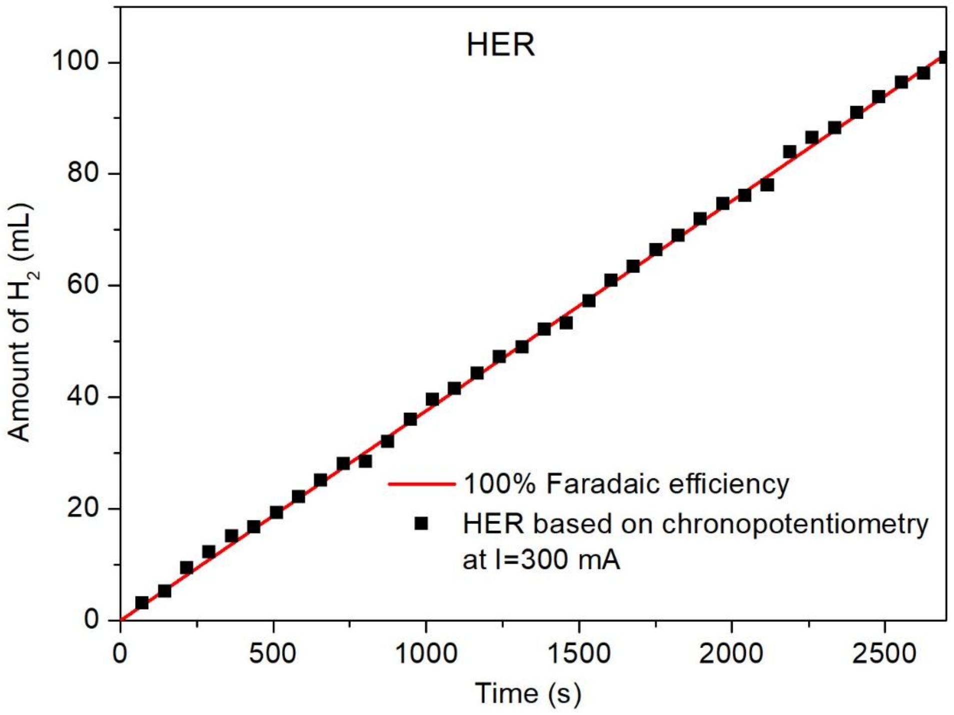

Figure S48. Determination of the Faradaic efficiency for the hydrogen evolution reaction in 3 M H_2SO_4 (anolyte ii (15 g $Fe_2(SO_4)_3 \cdot H_2O$ in 100 mL of 0.125 M H_2SO_4). Working electrode: Pt wire (A=3 cm^2); counter electrode Pt wire (A=2 cm^2). T=293 K. See also Figure S41.

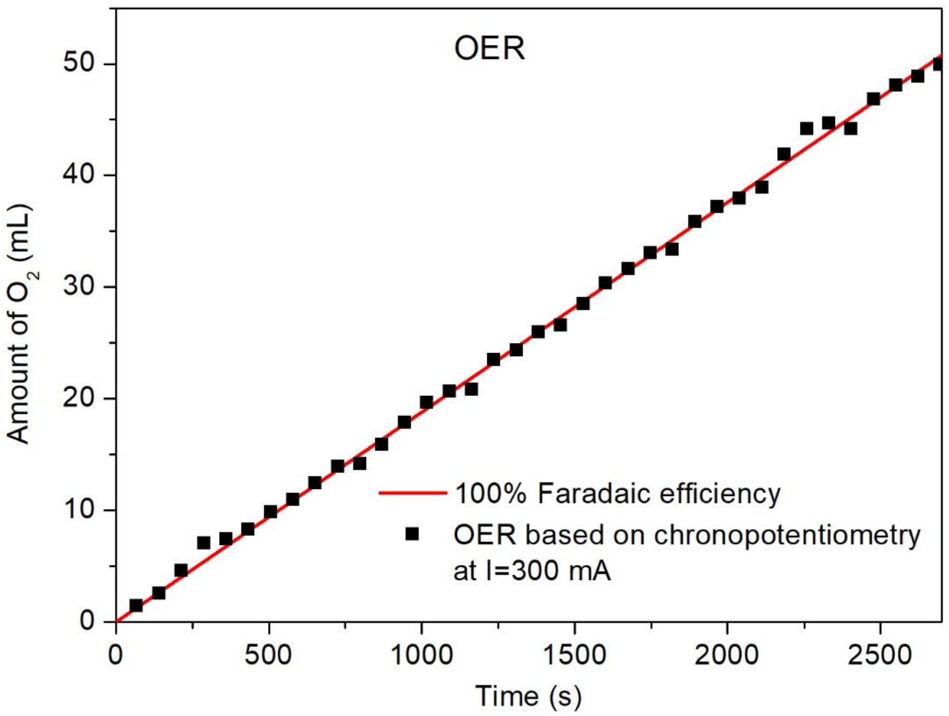

Figure S49. Determination of the Faradaic efficiency for the oxygen evolution reaction in anolyte ii (15 g $Fe_2(SO_4)_3 \cdot H_2O$ in 100 mL of 0.125 M H_2SO_4 (catholyte :3 M H_2SO_4). Working electrode: Pt wire (A=3 cm^2); counter electrode Pt wire (A=2 cm^2). T=293 K. See also Figure S41.

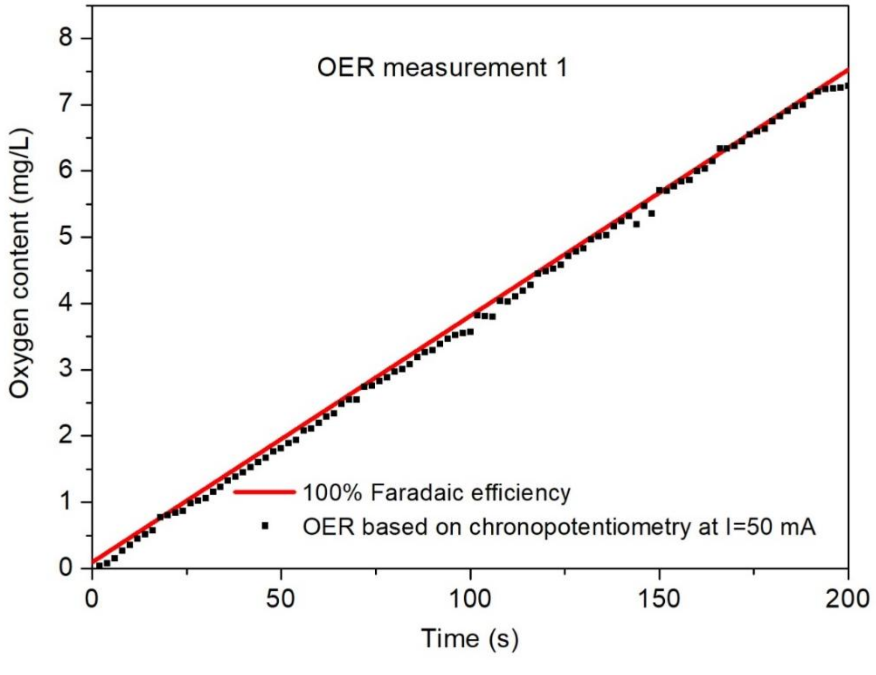

Figure S50. Determination of the Faradaic efficiency (measurement 1) for the oxygen evolution reaction in anolyte ii (15 g $Fe_2(SO_4)_3 \cdot H_2O$ in 100 mL of 0.125 M H_2SO_4 (catholyte:3 M H_2SO_4). Working electrode: Pt wire (A=3 cm^2); counter electrode Pt wire (A=2 cm^2) upon usage of the fluorescence quenching method. The current was set at 50 mA. Linear equation at 100% Faraday efficiency: OER: $y=3.768 \cdot 10^{-2} \cdot x + 0.09$ where x (s) is the measurement time and y is the oxygen content (mg/L). Faradaic efficiency=99.5% (t=200s).

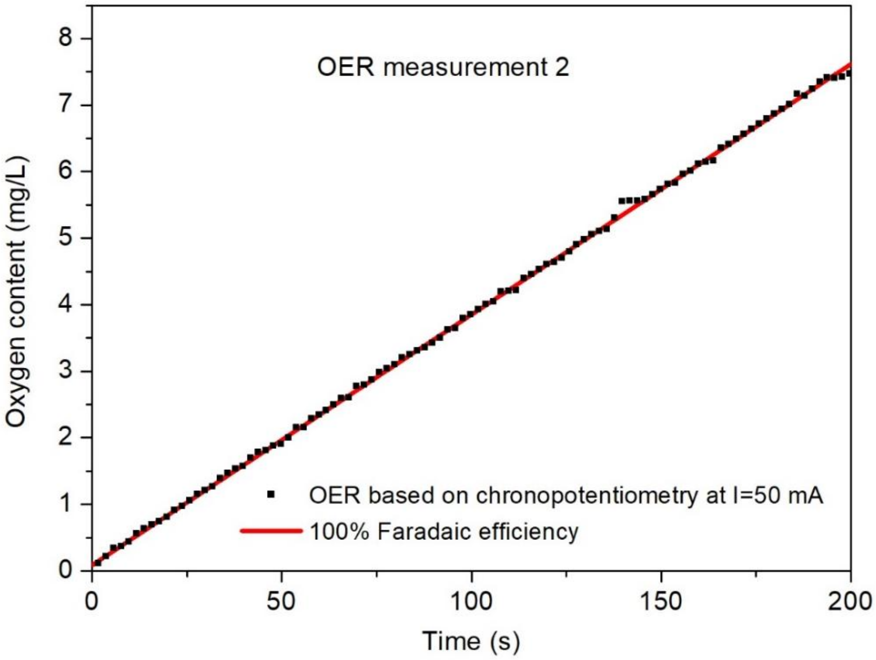

Figure S51. Determination of the Faradaic efficiency (measurement 2) for the oxygen evolution reaction in anolyte ii (15 g $Fe_2(SO_4)_3 \cdot H_2O$ in 100 mL of 0.125 M H_2SO_4 (catholyte:3 M H_2SO_4). Working electrode: Pt wire (A=3 cm^2); counter electrode Pt wire (A=2 cm^2) upon usage of the fluorescence quenching method. The current was set at 50 mA. Linear equation at 100% Faraday efficiency: OER: $y=3.768 \cdot 10^{-2} \cdot x + 0.08$ where x (s) is the measurement time and y is the oxygen content (mg/L). Faradaic efficiency=99.4% (t=200s).

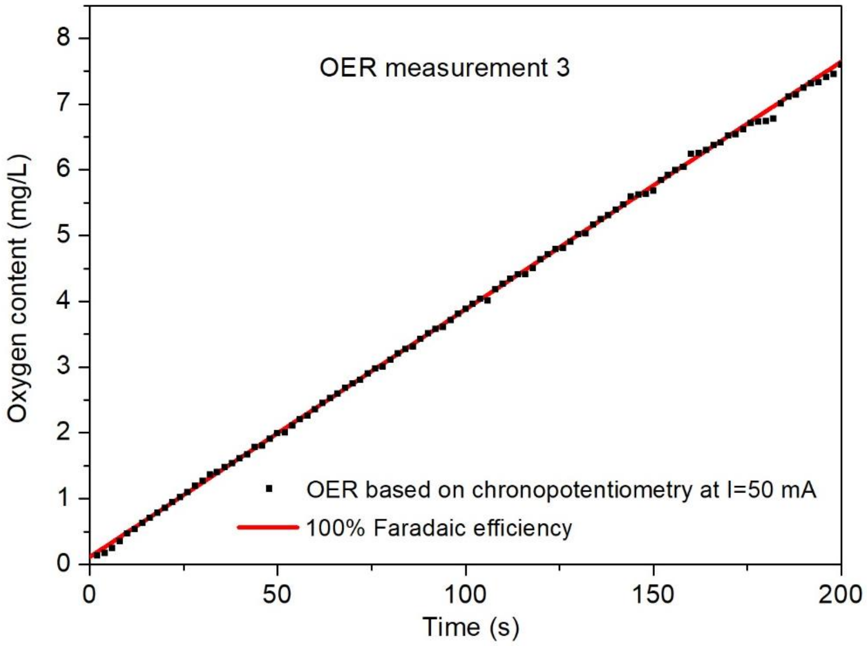

Figure S52. Determination of the Faradaic efficiency (measurement 2) for the oxygen evolution reaction in anolyte ii (15 g $Fe_2(SO_4)_3 \cdot H_2O$ in 100 mL of 0.125 M H_2SO_4 (catholyte:3 M H_2SO_4). Working electrode: Pt wire (A=3 cm^2); counter electrode Pt wire (A=2 cm^2) upon usage of the fluorescence quenching method. The current was set at 50 mA. Linear equation at 100% Faraday efficiency: OER: $y=3.768 \cdot 10^{-2} \cdot x + 0.11$ where x (s) is the measurement time and y is the oxygen content (mg/L). Faradaic efficiency=99.5% (t=200s).

References

[1] W. Kohn and L. J. Sham, *Phys. Rev*. **140**, A1133-A1138 (1965).
[2] G. Kresse and J. Hafner, *Phys. Rev. B* **47**, 558-561 (1993).
[3] G. Kresse and J. Furthmüller, *Phys. Rev. B* **54**, 11169-11186 (1996).
[4] G. Kresse and J. Hafner, *Phys. Rev. B* **49**, 14251 -14269 (1994).
[5] G. Kresse and D. Joubert, Phys. Rev. B **59**, 1758-1775 (1999).
[6] V. I. Anisimov, J. Zaanen, and O. K. Andersen, *Phys. Rev. B* **44**, 943-954 (1991).
[7] John P. Perdew, Kieron Burke, and Matthias Ernzerhof, *Phys. Rev. Lett.* **77**, 3865-3868 (1997).
[8] S. Grimme, J. Antony, S. Ehrlich, H. Krieg, *J. Chem. Phys.* **132** (15): 154104 (2010).
[9] R. Dronskowski, P. E. Blechl, J. Phys. Chem. **97**, 33, 8617–8624 (1993)
[10] A. A. Naik, K. Ueltzen, C. Ertural, A. J. Jackson and J. George, *J. Open Source Softw.* **9** (94), 6286 (2024).
[11] P. V. Prelesnik, F. Gabela, B. Ribar, I. Krstanovic, *Cryst Struct Comm*. **2**, 581-583 (1973).
[12] W. H. Baur, *Acta Cryst.* **17**, 1167- 1174 (1964).
[13] L.D. Iskhakova, V.K. Trunov, T.M. Shchegoleva, V.V. Ilyukhin, A.A. Vedernikov, *Kristallografiya*, **28**,651-657 (1983).
[14] T. Bookholt, X. Qin, B. Lilli, D. Enke, M. Huck, D. Balkenhohl, K. Rüwe, J. Brune, J. Klare, K. Küpper, A. Schuster, J. Bergjan, M. Steinhart, H. Gröger, D. Daum, H. Schäfer, *Small*, **20**, 2310665 (2024).
[15] H. Schäfer, S. Sadaf, L. Walder, K. Kuepper, S. Dinklage, J. Wollschläger, L. Schneider, M. Steinhart, J. Hardege, D. Daum, *Energy Environ. Sci.* **8**, 2685-2697 (2015).
[16] T. Bookholt, X. Qin, B. Lilli, D. Enke, M. Huck, D. Balkenhohl, K. Rüwe, J. Brune, J. Klare, K. Küpper, A. Schuster, J. Bergjan, M. Steinhart, H. Gröger, D. Daum, H. Schäfer, *Small*, **20**, 2310665 (2024).
